# Ultrasmall Fe_3_O_4_ nanoparticles self-assembly induced dual-mode T_1_/T_2_-weighted magnetic resonance imaging and enhanced tumor synergetic theranostics

**DOI:** 10.1038/s41598-024-59525-2

**Published:** 2024-05-09

**Authors:** Qinghua Xie, Xuemei Wang, Gaorui Zhang, Dawei Zhou, Yuxuan Zhao, Hong Liu, Jiazhi Duan, Dexin Yu, Yuanhua Sang

**Affiliations:** 1 State Key Laboratory of Crystal Materials, Shandong University, Jinan, 250100 China; 2 Department of Radiology, Qilu Hospital of Shandong University, Jinan, 250012 Shandong China; 3 Translational Medicine Research Center in Nano Molecular and Functional Imaging of Shandong University, Jinan, 250100 China; 4 Shandong BIOBASE Biology Co., Ltd, Jinan, 250000 Shandong China; 5 Qingzhou Peoples`S Hospital, Qingzhou, 262500 Shandong China

**Keywords:** Biomaterials, Nanobiotechnology

## Abstract

Individual theranostic agents with dual-mode MRI responses and therapeutic efficacy have attracted extensive interest due to the real-time monitor and high effective treatment, which endow the providential treatment and avoid the repeated medication with side effects. However, it is difficult to achieve the integrated strategy of MRI and therapeutic drug due to complicated synthesis route, low efficiency and potential biosafety issues. In this study, novel self-assembled ultrasmall Fe_3_O_4_ nanoclusters were developed for tumor-targeted dual-mode T_1_/T_2_-weighted magnetic resonance imaging (MRI) guided synergetic chemodynamic therapy (CDT) and chemotherapy. The self-assembled ultrasmall Fe_3_O_4_ nanoclusters synthesized by facilely modifying ultrasmall Fe_3_O_4_ nanoparticles with 2,3-dimercaptosuccinic acid (DMSA) molecule possess long-term stability and mass production ability. The proposed ultrasmall Fe_3_O_4_ nanoclusters shows excellent dual-mode T_1_ and T_2_ MRI capacities as well as favorable CDT ability due to the appropriate size effect and the abundant Fe ion on the surface of ultrasmall Fe_3_O_4_ nanoclusters. After conjugation with the tumor targeting ligand Arg-Gly-Asp (RGD) and chemotherapy drug doxorubicin (Dox), the functionalized Fe_3_O_4_ nanoclusters achieve enhanced tumor accumulation and retention effects and synergetic CDT and chemotherapy function, which serve as a powerful integrated theranostic platform for cancer treatment.

## Introduction

Various well-designed functional materials have been employed as the longitudinal relaxation time (T_1_) or transversal relaxation time (T_2_) weighted MRI contrast agents (CAs) for improving image contrast and sensitivity: T_1_ positive agents of paramagnetic species with bright signals^1,2^ and T_2_ negative agents of superparamagnetic particles with dark signals^3^. Each MRI mode has its advantage, for example, T_1_ weighted MRI possesses excellent resolution in displaying normal soft-tissue anatomy and T_2_ weighted MRI possesses more outstanding in soft tissue imaging with high sensitivity to detect lesions such as tumors and inflammation. However, single MRI mode cannot meet the high diagnostic requirements owing to their inherent defects^4^. Since each single imaging mode has unique strengths and limitations, the utilization of advantage combinations and defect elimination of both T_1_ and T_2_ imaging technologies to increase the target-to-background signal ratio (TBR) must be urgently developed to accurately locate and diagnose lesions^5,6^. Paramagnetic metal ions with high electron spin angular momentum such as Mn^2+^, Fe^3+^ and Gd^3+^ decrease the T_1_ of water protons, which efficiently modify the T_1_ mode MRI^7^. The T_2_ is sensitive to pathological and physiological stimuli with high magnetization. Generally, researchers mainly construct T_1_/T_2_ dual-mode MRI with individual T_1_ and T_2_ contrast agents^8–10^. Unfortunately, these composite structures may suffer from elaborate synthetic procedures and unsatisfactory interference of the relaxation process. Therefore, individual contrast agents with dual-mode MRI responses have attracted extensive interest^11,12^.

For the design of dual-mode T_1_/T_2_ MRI contrast agent, the Fe_3_O_4_ nanoparticles with tunable MRI ability are the mostly used materials. Ultrasmall Fe_3_O_4_ nanoparticles with diameters below 5 nm have been recognized to possess T_1_-enhancing contrast agents due to the abundant Fe^3+^ on the surface of nanoparticles, while their T_2_ effect decreases^13,14^. Usually, smaller Fe_3_O_4_ nanoparticles can enhance the T_1_ mode detection, and larger Fe_3_O_4_ nanoparticles can improve the T_2_ mode image due to their large innate high magnetic moment. The small size increases the surface-to-volume ratio of Fe ions, which improves their interaction with the surrounding water protons. The decrease in the magnetic core of superparamagnetic Fe_3_O_4_ nanoparticles weakens the T_2_-weighted MRI response^15,16^. To enhance the T_2_ effect, self-assembled ultrasmall Fe_3_O_4_ nanoparticles were proposed to increase the magnetization. Therefore, some recent studies elaborately designed the surface chemistry of ultrasmall Fe_3_O_4_ nanoparticles to make the particle size adjustable with some bioconditions, including specific pH^17,18^, hypoxia^19^, glutathione^20–23^ and light^24^. However, some limitations still remain, such as complicated synthesis procedures, small production, and limited application conditions. Moreover, it is difficult to achieve homogeneity via the self-assembly of ultrasmall Fe_3_O_4_ nanoparticles, which affects the signal reliability. More importantly, some designs enable T_1_ to T_2_ conversion but fail to simultaneously possess both T_1_ and T_2_ effects, which urges more devotion to developing ultrasmall Fe_3_O_4_ nanoparticle-based T_1_/T_2_-weighted MRI contrast agents. Nanoparticles smaller than 5 nm can be facially cleared by the kidney, those 10–20 nm in size can be taken up rapidly, and the half-lifetime of the contrast agent in blood circulation was low^25,26^. The small size would make it easier for the nanoparticles to enter the tumor by permeation and retention effects, which would be beneficial for MRI examination. Therefore, the size control of the Fe_3_O_4_ contrast agent should be considered an important factor.

Integrating high bioimaging capability and therapeutic efficacy simultaneously show promising perspective for developing new effective theranostic agent^27,28^. The ultrasmall size of Fe_3_O_4_ nanoparticles not only endow the excellent MRI ability, but also enhance the exposed Fe ion content, which is essential for the CDT of tumor. The Fe^2+^ on the surface of ultrasmall Fe_3_O_4_ nanoparticles could serve as an effective catalyzer of high content H_2_O_2_ in the tumor tissue to produce poison OH·, which could effectively induce apoptosis of cancer cell. Chemotherapy, one of the most used cancer treatments in clinic, suffers from no targeting and drug resistance. The collaborative effect of ultrasmall Fe_3_O_4_ nanoparticles and chemotherapeutics could endow the MRI guided synergetic CDT and chemotherapy^29,30^. Moreover, integrated cancer cell targeting molecule could overcome the shortcoming of no targeting of therapeutic drugs. Therefore, the design and preparation of ultrasmall Fe_3_O_4_ nanoparticle based nanomedicine for constructing tumor-targeted imaging and treatment nanoplatform in one system hold huge promising for resolving obstacle of tumor elimination^31^.

**Scheme 1:**
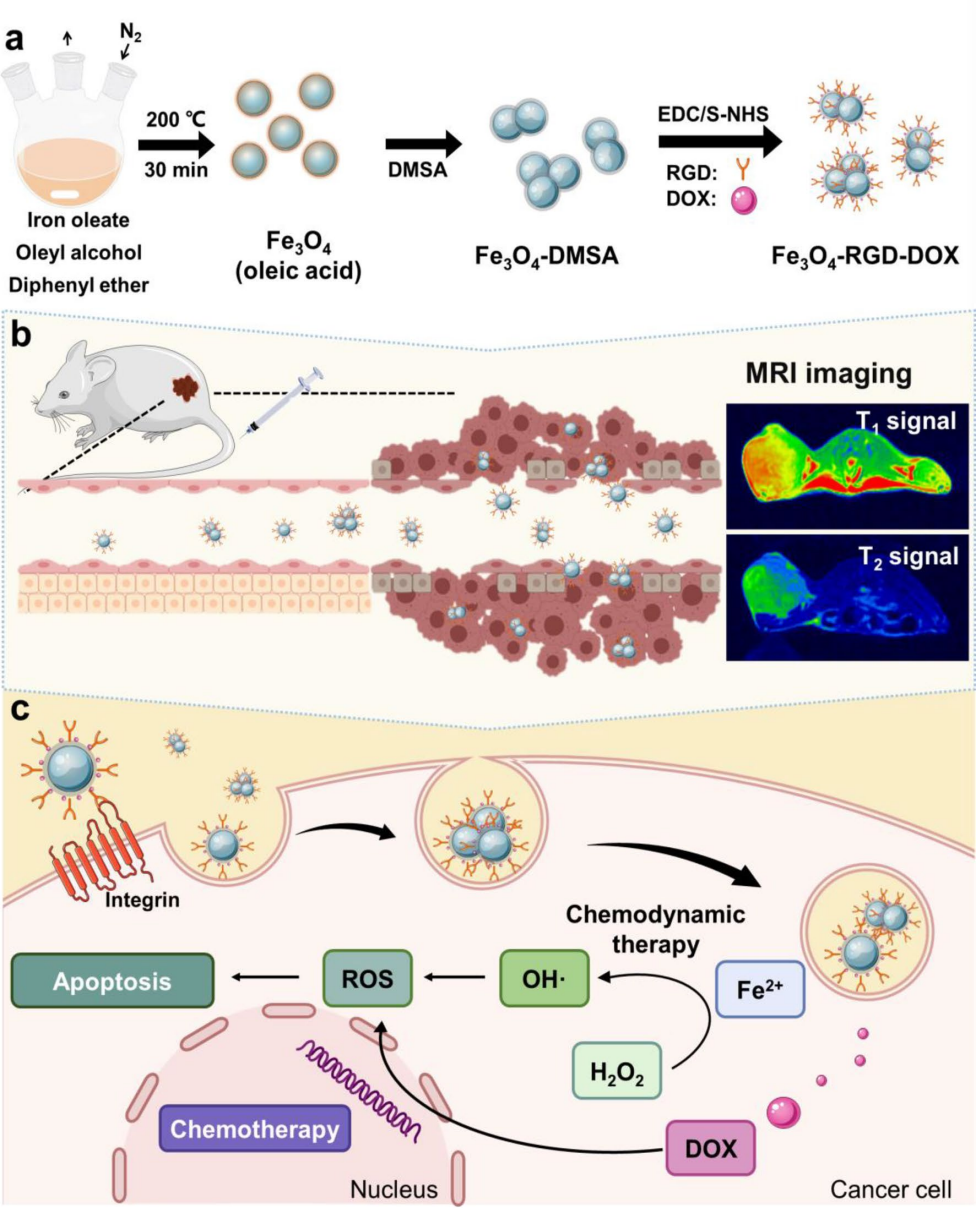
Schematic of ultrasmall Fe_3_O_4_ nanoparticle-based nanoclusters for tumor-targeted dual-mode T_1_/T_2_-weighted MRI and synergetic CDT and chemotherapy.

In the current work, we synthesized highly crystalline ultrasmall Fe_3_O_4_ nanoparticle-based self-assembled nanoclusters with mass production for tumor-targeted dual-mode T_1_/T_2_-weighted MRI guided synergetic CDT and chemotherapy (Scheme 1). The ultrasmall Fe_3_O_4_ nanoparticles were synthesized with a size of approximately 4 nm using a solvothermal method. After modification with 2,3-dimercaptosuccinic acid (DMSA), ultrasmall Fe_3_O_4_ nanoparticles (Fe_3_O_4_-DMSA) can homogenously self-assemble into nanoclusters with several particles and maintain excellent dispersity and colloidal stability^32^. The Fe_3_O_4_-DMSA nanoclusters show excellent dual-mode T_1_/T_2_ MRI property due to the favorable size distribution and could effectively induce apoptosis of cancer cell by CDT. Further conjugation with Arg-Gly-Asp (RGD) and doxorubicin (DOX), RGD ligand shows an improved binding affinity toward 4T1 cells through an overexpression of α_v_β_3_ receptors^33^. Dox is an effective chemotherapy drugs for cancer cell. The treatment of self-assembled ultrasmall Fe_3_O_4_ nanoclusters conjugated with RGD and DOX shows efficient T_1_/T_2_ dual-mode MRI guided tumor-targeted synergetic CDT and chemotherapy. Therefore, this work provides a novel and convenient method to prepare integrated tumor-targeted MRI and nanodrugs treatment as well as proposes the concept of nanocluster-based functional MRI contrast agents and integrated treatment platform.

## Experiential sections

### Materials

FeCl_3_·6H_2_O, sodium carbonate, sodium oleate, diphenyl ether, oleyl alcohol, doxorubicin (Dox) and 2,3-dimercaptosuccinic acid were purchased from Shanghai Macklin Biochemical Co., Ltd (Shanghai). Ethanol, n-hexane, and tetrahydrofuran were purchased from Sinopharm Chemical Reagent Co., Ltd. Arg-Gly-Asp was obtained from Meilun Biotechnology Co., Ltd. (Dalian). 1-(3-Dimethylaminopropyl)-3-ethylcarbodiimide hydrochloride (EDC) and sulfo-N-hydroxysuccinimide (S-NHS) were obtained from Shanghai Yuanye Bio-Technology Co., Ltd. Aminofluorescein was obtained from Shanghai Aladdin Bio-Chem Technology Co., Ltd. Propidium iodide (PI), calcein-AM, and Cell Counting Kit-8 were obtained from Dojindo Laboratories, Japan. The Perls Stain Kit and ROS fluorescence probe (2,7-dichlorodihydrofluorescein diacetate, DCFH-DA) were from Beijing Solarbio Science & Technology Co., Ltd.

### Instrument and characterization

The morphology of synthesized Fe_3_O_4_ ultra-small nanoparticles and Fe_3_O_4_-DMSA nanoclusters was characterized by transmission electron microscopy (TEM; JEM-2100, JEOL, Tokyo, Japan), and the crystal structure of the Fe_3_O_4_ nanoparticles was characterized by high-resolution transmission electron microscopy (HRTEM, JEM-2100, JEOL, Tokyo, Japan) and X-ray diffraction (XRD) patterns (D8 Advance, Bruker, Ettlingen, Germany). The element composition and atomic valence state were characterized by X-ray photoelectron spectroscopy (XPS). Fourier transform infrared (FTIR) spectra of the obtained nanomaterials were recorded by an IR spectrophotometer (Nicolet Nexus 670, Thermo Fisher Scientific, Inc., Waltham, MA). Ultraviolet–visible (UV–vis) light absorption spectra were obtained from a UV–vis spectrophotometer (UV-6100, Meipuda, Xi’an, China). The zeta potential and size distribution of the synthesized nanoparticles were characterized by a Malvern Zetasizer Nano Series. Element quantification was performed by an X-ray electron probe microanalyzer (EPMA). The ion concentration was quantified by inductively coupled plasma-mass spectrometry (ICP-MS). The saturation magnetization of Fe_3_O_4_ nanoparticles was measured by a vibrating sample magnetometer (MicroMagTM Model 2900 AGM system). The relaxation rate of MRI images of the Fe_3_O_4_ nanoprobe was tested by a 3.0-T clinical MRI scanner (GE Signa HDx 3.0 T MRI, USA) and a 16-channel brain coil.

### Mass production, modification and conjugation of ultra-small Fe_3_O_4_ nanoparticles

The mass-production method of ultra-small Fe_3_O_4_ nanoparticles was adapted from a previous report^34^. Briefly, all iron-oleate was obtained according to previous report^35^. Then, iron-oleate was added to 200 mL diphenyl ether and 50 mL oleyl alcohol. The mixture was heated to 200 °C and maintained for 30 min under N_2_ atmosphere. The product was washed with ethanol and dried at 60 °C. The obtained brown powder was ultra-small Fe_3_O_4_ nanoparticles.

Then, the nanoparticles were modified with DMSA molecules to synthesis Fe_3_O_4_-DMSA nanoclusters. Briefly, 20 mg of Fe_3_O_4_ nanoparticles, 30 mg of DMSA, and 15 mg of Na_2_CO_3_ were added to a mixture of tetrahydrofuran and water and ultrasonicated for 30 min. Afterwards, the mixture was collected by centrifugation and freeze-dried to obtain Fe_3_O_4_-DMSA nanoclusters. Fe_3_O_4_-DMSA nanoclusters can be mass-produced by amplifying this process tween times.

To conjugate the RGD ligand to synthesis Fe_3_O_4_-RGD nanoclusters, 4 mg aqueous Fe_3_O_4_-DMSA was added to the mixture of 1 mg of RGD, 2 mM EDC, and 4 mM S-NHS and reacted at 4 °C overnight. The product was dialyzed for 24 h. To synthesis Fe_3_O_4_-RGD-DOX, additional 0.2 mg of DOX was added to the same action media as RGD and the processing procedure was the same as the conjugation of RGD ligand. The aminofluorescein conjugation procedure was identical to the RGD. The bonding mechanism between Fe_3_O_4_ and RGD as well as DOX are through the esterification between the COOH of Fe_3_O_4_-DMSA and NH_2_ of DOX molecule and RGD ligand.

### T_1_ and T_2_ relaxivity measurement of Fe_3_O_4_-DMSA nanoclusters

The T_1_ and T_2_ relaxivity test of the Fe_3_O_4_-DMSA or Fe_3_O_4_-RGD solution were performed using a 3.0 T MR scanner (GE Signa HDx 3.0 T MRI, USA). The Fe_3_O_4_-DMSA aqueous with different concentrations (0, 2000, 1000, 500, 250, 125, 62.5, 31.2, 15.6, 7.8 and 3.9 μg/mL) was measured. The following parameters were adopted in the data acquisition procedure: T_1_-weighted images: echo time (TE) = 20 ms; repeat time (TR) = 640 ms; FOV = 14 × 14 cm^2^; matrix = 256 × 256; slice thickness = 1.5 mm; spacing = 0.2 mm. (b), T_2_-weighted images: TE = 90 ms; TR = 4000; FOV = 14 × 14 cm^2^; matrix = 256 × 256; slice thickness = 1.5 mm; spacing = 0.2 mm. (c), T_1_ mapping: TR = 4000, 3000, 2000, 1500, 1250, 1000, 750, 500 ms; TE = 20 ms; (d), T_2_ mapping: TR = 3000 ms, TE = 20, 40, 60, 80, 100 and 120 ms; The reciprocal of T_1_ and T_2_ relaxation times plotted against the Fe concentrations and transverse relaxivity (r_1_/r_2_) was obtained by a linear fit.

### Cell lines and animal model

4T1 (mouse breast cancer) was obtained from the Institute of Biochemistry and Cell Biology (IBCB, Shanghai, China). The cells were cultured in RPMI-1640 culture medium with 10% fetal bovine serum (Gibco, USA) and 0.1% penicillin–streptomycin (Gibco, USA). Then, they were cultured in a humidified atmosphere at 37 °C in an incubator with 5% CO_2_.

All animal were purchased from the Jinan Peng Yue Laboratory Animal Co., Ltd, Jinan, China. The mice were fed with a standard laboratory diet and water. All mouse research were conducted in line with protocols approved by the Laboratory Animal Ethical and Welfare Committee of Shandong University Cheeloo College of Medicine, China (accreditation number: SYXK:20190005), and all animal experiments methods were performed according to these guidelines and regulations. To develop the 4T1 bearing tumor model, BALB/c male mice (6 to 8 week-old) were orthotopically injected with 1 × 10^6^ 4T1 cells in the right hind leg.

### Tumor cell targeting assay of Fe_3_O_4_-RGD nanoclusters in vitro

To verify the RGD targeting effect, Fe_3_O_4_-DMSA and Fe_3_O_4_-RGD were added to cultured 4T1 cells at 100 μg/mL for 2 h. Then, the cancer cells were fixed with 2.5% glutaraldehyde and dehydrated in 30%, 50%, 70%, 80%, 90%, and 100% ethanol. The iron element content and distribution of the cells in each group were analyzed by EPMA. Additionally, Fe_3_O_4_-DMSA and Fe_3_O_4_-RGD were conjugated with aminofluorescein and added to the cultured cells at a concentration of 100 μg/mL for 2 h. The fluorescence intensity of each group at 568 nm was observed by laser scanning confocal microscopy (LSCM, FV 300, Olympus, Japan). To verify that cellular uptake was receptor-specific, competition experiments were conducted using flow cytometry. The 4T1 cells were plated at 5 × 10^5^ cells/ml in a 6-well plate and incubated with aminofluorescein-labeled Fe_3_O_4_-DMSA or Fe_3_O_4_-RGD solution. The cells were incubated for 12 h, washed twice with PBS, trypsinized, centrifuged and resuspended in 500 μl PBS before a flow cytometry analysis using a FACS Calibur flow cytometer (Becton Dickinson, NJ, USA).

Prussian blue staining kit were utilized to further detecte the RGD targeted effect for 4T1 cell uptake. Briefly, 4T1 cells were seeded in 24-well plates and incubated overnight. After washing with PBS twice, the cells were incubated with Fe_3_O_4_-DMSA (50 μg/mL, 100 μg/mL and 150 μg/mL) and Fe_3_O_4_-RGD (50 μg/mL, 100 μg/mL and 150 μg/mL). After 12 h cells were washed three times. Cells were fixed for 15 min in 4% paraformaldehyde, and then incubated for 25–30 min with 10% potassium ferrocyanide, rewashed twice with PBS, and counter stained with nuclear fast red for 10 min. Cells containing intracytoplasmic blue granules were defined to be Prussian blue staining positive.

### Dual-mode T_1_/T_2_-weighted MR imaging ability of Fe_3_O_4_-DMSA and Fe_3_O_4_-RGD nanoclusters in vitro and vivo

For in vitro MRI, the 4T1 cells were washed with PBS and resuspended in 500 μL cell culture medium to verify the MRI effect at the cellular level by 3.0 T MRI scanner. T_1_-weighted imaging was performed with the following parameters: matrix size = 256 × 256, TR = 1200 ms, TE = 20 ms, slice thickness = 0.8 mm; T_2_-weighted imaging: matrix size = 256 × 256; TR = 2000 ms, TE = 62 ms; slice thickness = 0.8 mm. The T_2_-weighted signal change was calculated by using the following formula: SIi/SIc × 100% (i = 0.5, 1, 2, and 4 h), where SIc and SIi were the signal intensities of the 4T1 cells before and 0.5, 1, 2, and 4 h after incubation with Fe_3_O_4_-DMSA or Fe_3_O_4_-RGD, respectively. Additionally, the cultured cells in each group were stained with Prussian blue after 12 h of incubation with Fe_3_O_4_-DMSA and Fe_3_O_4_-RGD according to the manufacturer instructions to confirm the RGD-enhanced targeting effect.

To verify the in vivo MRI effect, 4T1 tumor-bearing mice were intravenous injection of Fe_3_O_4_-DMSA or Fe_3_O_4_-RGD (5 mg Fe/kg). T_1_-weighted and T_2_-weighted MRI of 4T1 tumor-bearing mice were acquired on a 3.0 T scanner. Scanned with the following parameters: T_2_ weighted imaging; FOV (field of view) = 12 × 12 cm^2^; matrix size = 256 × 256; slice thickness = 1 mm; TE = 60 ms; TR = 2000 ms; NEX = 2; T_1_ weighted imaging; FOV (field of view) = 12 × 12 cm^2^; matrix size = 256 × 256; slice thickness = 1 mm; TE = 10 ms; TR = 500 ms; NEX = 2 s. The MR imaging of mice were obtained at pre-injection and at 1 h, 2 h, 4 h, 8 h and 12 h post-injection. After MR scanning, slices covering the entire tumor region were prepared. TEM, ICP-MS and Prussian blue stained were utilized to verify the MRI results.

### Biosafety evaluation of nanoparticles

To assess the hemolytic properties of Fe_3_O_4_-DMSA and Fe_3_O_4_-RGD nanomaterials, 2 mL of blood was dispersed to 4 mL of PBS (pH 7.4) and centrifuged at 1200 r/min for 10 min to isolate the red blood cells (RBCs). After washing with saline 3 times until the supernatant was colorless, the red blood cells were diluted with physiological saline to obtain 2% (v/v) red cell suspensions. Then, Fe_3_O_4_-DMSA and Fe_3_O_4_-RGD at different final concentrations (25, 50, and 100 μg/mL) were co-incubated with red cell suspensions for 1 h at 37 °C. Simultaneously, equal volumes of distilled water and physiological saline were selected as positive and negative controls, respectively. After the incubation, the absorbance was measured by a microplate reader at 570 nm. The hemolytic degree was expressed by the hemolytic ratio using the following formula: hemolysis ratio = (O. D of sample − O. D of negative control)/(O. D of positive control − O. D of negative control) × 100%.

### Fe_3_O_4_-RGD-DOX nanomedicine for T_1_/T_2_ dual-mode MR imaging and synergistic CDT and chemotherapy in vitro

The RGD targeted CDT effect of Fe_3_O_4_ nanoclusters was furtherly studied. 4T1 cells were treated by the co-incubation of Fe_3_O_4_-DMSA (2.5, 5, 10, 20, 50, 100 and 150 μg/mL) and Fe_3_O_4_-RGD (2.5, 5, 10, 20, 50, 100 and 150 μg/mL) for 24 h. Then, the cell viability was determined using the CCK-8 assay (n = 5). The 4T1 cells were seeded in 24 cell culture plates, and a designed concentration of Fe_3_O_4_-DMSA was added to the culture medium for a specific time. Live/dead staining was performed to evaluate the cytotoxicity of the nanoclusters. Additionally, ROS staining by DCFH-DA was performed to briefly demonstrate the reason for toxicity after the 2 h co-culture of Fe_3_O_4_ at 50 μg/mL, 150 μg/mL and cancer cells.

To further verify the synergistic chemotherapy and CDT induced by Fe_3_O_4_-RGD-DOX nanomedicine, 4T1 cells were exposed to different concentrations of Fe_3_O_4_-DMSA (100 μg/mL), Fe_3_O_4_-RGD (100 μg/mL), DOX (5 μg/mL) and Fe_3_O_4_-RGD-DOX (100 μg Fe_3_O_4_/mL, 5 μg DOX/mL) and further measured by Living/dead staining and CCK-8 test. Untreated cells in the medium were used as a control. Corresponding groups without cells were used as blanks. The cell viability was calculated by assuming 100% viability in the control cells. The proliferation ability was analyzed through an EdU Proliferation Kit. Briefly, 4T1 cells grown in 96-well plates were subjected to different concentrations of Fe_3_O_4_-DMSA (100 μg/mL), Fe_3_O_4_-RGD (100 μg/mL), DOX (5 μg/mL) and Fe_3_O_4_-RGD-DOX (100 μg Fe_3_O_4_/mL, 5 μg DOX/mL) for 24 h. Then EdU was incorporated into the treated cells and detected through a catalyzed reaction with a fluorescently labeled azide and were observed by fluorescence microscopy.

To further detected the iron oxide distribution in vitro, 4T1 cells were incubated with PBS, Fe_3_O_4_-DMSA (100 μg/mL), Fe_3_O_4_-RGD (100 μg/mL), DOX (5 μg/mL) and Fe_3_O_4_-RGD-DOX (100 μg Fe_3_O_4_/mL, 5 μg DOX/mL), after 12 h. Then Prussian blue staining were utilized to show positive 4T1 cells. The stain method were performed in agreement with previously described methods. Dichlorofluorescein diacetate (DCFH-DA) was used to verify the intracellular ROS. 4T1 cells were treated with different concentrations of Fe_3_O_4_-DMSA (100 μg/mL), Fe_3_O_4_-RGD (100 μg/mL), DOX (5 μg/mL) and Fe_3_O_4_-RGD-DOX (100 μg Fe_3_O_4_/mL, 5 μg DOX/mL) for 6 h. After that, the cells were washed with PBS and incubated with DCFH-DA solution (10 μM) for another 20 min. Afterwards, the cells were washed and then observed by CLSM. Mean intracellular fluorescence intensity was analyzed with the ImageJ software.

### Dual-mode T_1_/T_2_-weighted MR imaging and synergistic effect of RGD targeting CDT and chemotherapy in vivo

To confirm the effective of MRI and combination therapy of CDT and chemotherapy. T_1_ and T_2_ weighted MR imaging were detected before and after intravenous injection of 100 µL normal saline, Fe_3_O_4_-DMSA (5 mg/kg), Fe_3_O_4_-RGD (5 mg/kg), DOX (250 μg/kg) and Fe_3_O_4_-RGD-DOX (5 mg/kg Fe_3_O_4_/mL, 250 μg/kg Dox). The scanned parameters were performed in agreement with previously described methods. Coronal MRI data were collected pre-injection and post-injection.

### Statistical analysis

All data were expressed as the average ± standard deviation. Statistical comparison between two groups was analyzed by the Student`s t-test. Analysis of variance (ANOVA) was used to compare the differences in different groups, and the data were defined with *p < 0.05, **p < 0.01 and ***p < 0.001.

### Ethics statement

This study is reported in accordance with ARRIVE guidelines.

## Results and discussions

### Synthesis and characterizations of ultra-small Fe_3_O_4_ nanoparticles and Fe_3_O_4_ nanoclusters

**Figure 1:**
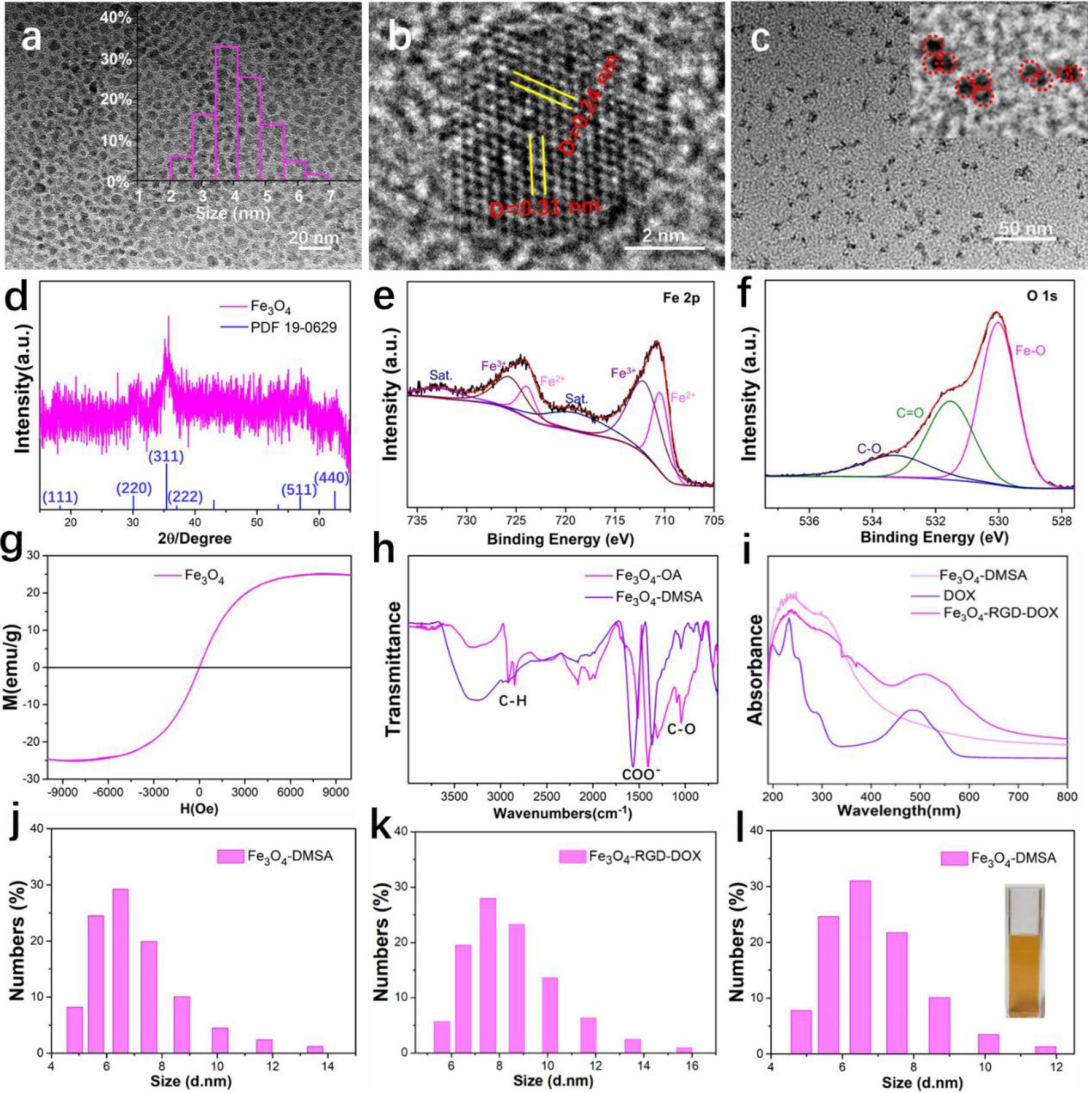
Characterization of ultra-small Fe_3_O_4_ nanoparticles and Fe_3_O_4_ nanoclusters. (**a**) TEM image of synthesized ultra-small Fe_3_O_4_ nanoparticles and the size distribution (inset). (**b**) HRTEM image of the ultra-small Fe_3_O_4_ nanoparticles. (**c**) TEM image of the synthesized Fe_3_O_4_-DMSA nanoclusters. (**d**) XRD pattern, (**e**,**f**) XPS pattern and (**g**) Saturation magnetization curve of ultra-small Fe_3_O_4_ nanoparticles. (**h**) FTIR spectra of Fe_3_O_4_ nanoparticles and Fe_3_O_4_-DMSA nanoclusters. (**i**) UV–vis patterns of Fe_3_O_4_-DMSA, DOX and Fe_3_O_4_-RGD-DOX. (**j**) Size distribution of Fe_3_O_4_-DMSA nanoclusters and (**k**) Fe_3_O_4_-RGD-DOX nanoclusters. (**l**) Size distribution of Fe_3_O_4_-DMSA nanoclusters after being placed at 4 °C for 10 months and a real image of the aqueous solution (inset).

The mass production of nearly 2 g in one reaction of the synthesized Fe_3_O_4_ nanoparticles is shown in Fig. S1, and the morphology was characterized by TEM (Figs. 1a, S2) and HRTEM (Figs. 1b, S3a). The obtained nanoparticles were of uniform shape, the size distribution was approximately 2–8 nm, and the main size was located at 4 nm (Fig. 1a, inset). The crystal plane spacings of 0.24 nm and 0.31 nm correspond to the (4 0 0) and (5 1 1) crystal planes, respectively, as shown in the HRTEM image. The well crystallinity of the individual ultra-small Fe_3_O_4_ nanoparticles is confirmed, which benefits the increased saturation magnetization. During the modification process with DMSA molecules, the Fe_3_O_4_ nanoparticles self-assemble into nanoclusters with 2–3 homogeneously embedded nanoparticles (Fig. 1c and the inset). Figure S4 confirms the stable synthesis and homogeneity of the Fe_3_O_4_-DMSA nanoclusters. The XRD pattern (Fig. 1d) of synthesized ultra-small Fe_3_O_4_ nanoparticles confirms the standard Fe_3_O_4_ phase (standard PDF card: 19-0629). The weak diffraction peak was assigned to the small size of the as-synthesized nanoparticles. Moreover, the synthesized nanoparticles were further analyzed by XPS (Fig. 1e,f). The spectrum of Fe 2p clearly shows the peaks of Fe^2+^ and Fe^3+^, which confirms the composition of Fe_3_O_4_^36^. The spectrum of O 1s contains the peaks assigned to Fe–O, C=O, and C–O, which indicates the coexistence of Fe_3_O_4_ and oleic acid molecules. This is the key reason for the hydrophobic property of the as-synthesized Fe_3_O_4_ nanoparticles and the requirement of DMSA surface modification. The saturation magnetization curve (Fig. 1g) measured by a vibrating sample magnetometer at room temperature confirms the superparamagnetic property of the obtained ultra-small Fe_3_O_4_ nanoparticles with a large saturation magnetization (25 emu/g). The modification of Fe_3_O_4_ with DMSA molecules was studied by FTIR spectroscopy (Fig. 1h). The broad peaks at 3297 cm^−1^ and 3252.5 cm^−1^ correspond to the stretching vibrations of O–H for oleyl alcohol and DMSA, respectively. The peaks at 1516.6 and 1402 cm^−1^ correspond to the symmetric and asymmetric stretches of carboxylate (COO–) in oleyl acid, while the peaks at 1567.4 and 1361 cm^−1^ correspond to the COO– in DMSA. All C–O stretching vibrations in oleyl alcohol and DMSA are at approximately 1045 cm^−1^. The decreased C–H stretching vibrations at 2916.5 and 2848.1 cm^−1^ of oleyl alcohol indicate the substitution of DMSA at the surface of Fe_3_O_4_. Notably, the as-synthesized Fe_3_O_4_ nanoparticles are spontaneously surface-modified by the organic solvent, which contributes to the good dispersion of Fe_3_O_4_ nanoparticles in the organic solvent (Fig. S5). The surface hydrophilic treatment of nanoparticles is essential for the bio-application. XPS spectra of the Fe_3_O_4_-DMSA nanoclusters were also obtained, and the existence of S indicates surface modification by DMSA (Fig. S6). DOX molecule was confirmed to be conjugated on the surface of Fe_3_O_4_ nanoclusters in the UV–vis absorbance result to constitute the nanomedicine (Fig. 1i). The size distribution of Fe_3_O_4_-DMSA is approximately 4.5–13.5 nm and the main size locates at 6 nm. (Fig. 1j). The conjugation of the RGD ligand and DOX molecule does not change the trend of the size distribution but increases the size by approximately 2 nm (Fig. 1k). Notably, the Fe_3_O_4_-DMSA nanoclusters show excellent colloidal stability. The nanocluster suspension in water remains clear and steady after being placed at 4 °C for 10 months and the size distribution (Fig. 1l) is identical to that of before.

### Tumor cell targeting ability of Fe_3_O_4_-RGD nanoclusters

**Figure 2:**
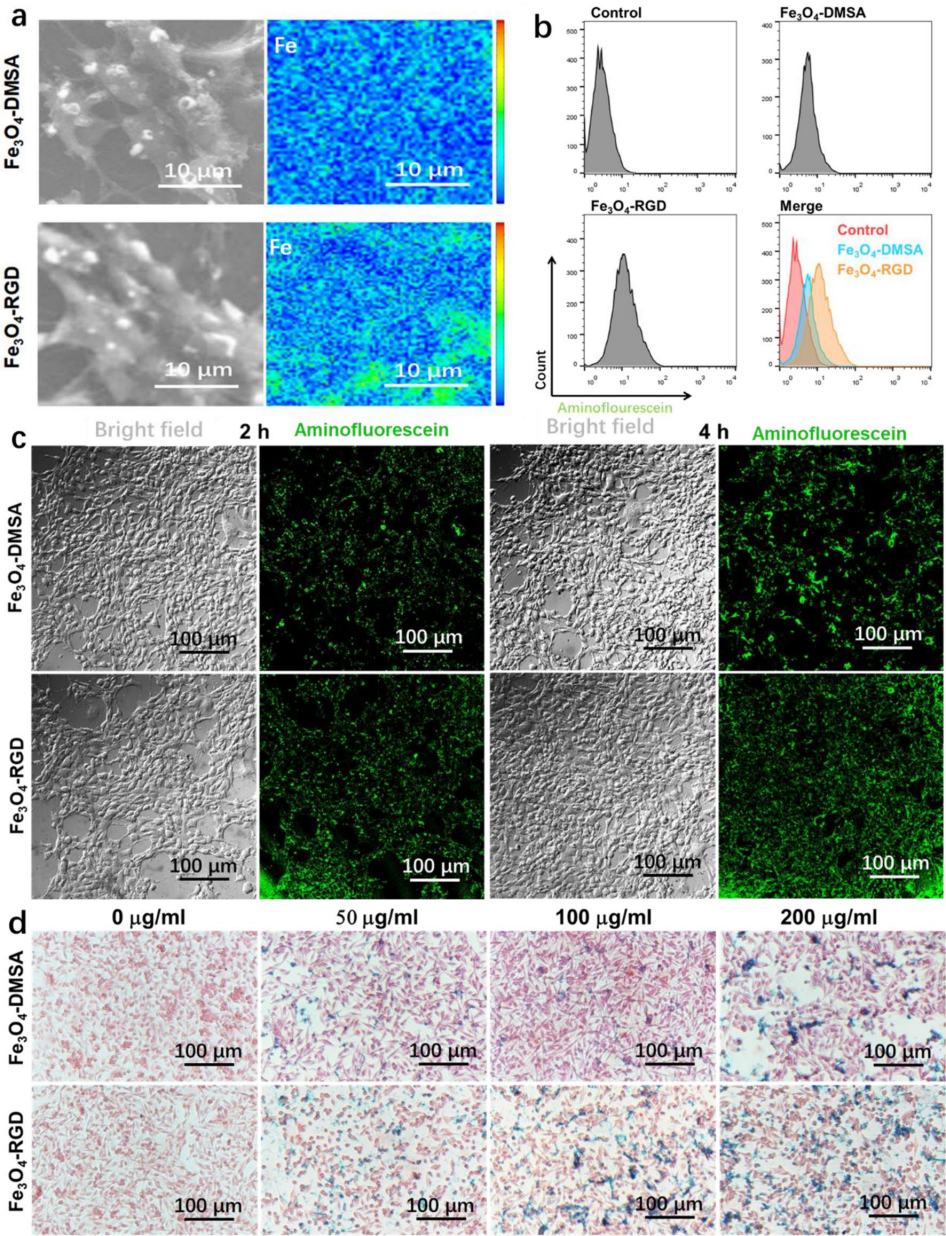
Tumor cell targeting ability of Fe_3_O_4_-RGD. (**a**) Iron element content distribution on the surface of 4T1 cells after being cultured with Fe_3_O_4_-DMSA and Fe_3_O_4_-RGD nanoclusters (color scale bar shows the Fe element content). (**b**) Flow cytometry assay of 4T1 cells after incubation with fluorescence labeled Fe_3_O_4_-DMSA and Fe_3_O_4_-RGD nanoclusters. (**c**) Fluorescence labeling of 4T1 cells with Fe_3_O_4_-aminofluorescein and Fe_3_O_4_-RGD-aminofluorescein for 2 h. (**d**) Prussian blue staining of 4T1 cells after being cultured with different concentrations of Fe_3_O_4_-DMSA and Fe_3_O_4_-RGD nanoclusters for 12 h.

Nanomaterials usually possess passive targeting ability in tumor tissue due to the enhanced permeation and retention effect (EPR) and transport effect mediated by endothelial cells^37^. This ability is beneficial for the designed Fe_3_O_4_-DMSA nanocluster. However, due to the low half-lifetime of the contrast agent in blood circulation, passive targeting is limited. The active targeting ability of Fe_3_O_4_-DMSA nanoclusters is endowed by conjugation with the tumor targeting RGD ligand. It is expected to improve the accumulation of Fe_3_O_4_ nanoclusters in the tumor region and realize a favorable contrast effect. As shown in Fig. 2a, the iron element content was analyzed by EPMA after the 4T1 cells were cultured with Fe_3_O_4_-DMSA and Fe_3_O_4_-RGD for 2 h. More iron was detected in the Fe_3_O_4_-RGD group than in the Fe_3_O_4_-DMSA group, which indicates the better accumulation property of Fe_3_O_4_-RGD nanoclusters. Furthermore, the aminofluorescein molecule was conjugated with the Fe_3_O_4_-DMSA and Fe_3_O_4_-RGD nanoclusters to locate the nanoparticles by fluorescence. As shown in Fig. 2b, the 4T1 cells were cocultured with fluorescence-labeled Fe_3_O_4_-DMSA and Fe_3_O_4_-RGD nanoclusters and assayed by flow cytometry. The results convey that more cells were labeled with fluorescence in the Fe_3_O_4_-RGD group than in the Fe_3_O_4_-DMSA group, which shows that the RGD ligand enhances the cell uptake ability. Then, fluorescence-labeled Fe_3_O_4_-DMSA and Fe_3_O_4_-RGD nanoclusters were cultured with 4T1 cells at a concentration of 100 μg/mL for 2 and 4 h. As shown in Fig. 2c, after the culture with Fe_3_O_4_-RGD, the green fluorescence intensities were higher than those cultured with Fe_3_O_4_-RGD for 2 and 4 h. This result confirms the enhanced targeting effect of the RGD ligand. Prussian blue staining was utilized to further study the existence of iron in 4T1 cells after culturing with Fe_3_O_4_-DMSA and Fe_3_O_4_-RGD nanoclusters at various concentrations for 12 h. As shown Fig. 2d, the staining results are consistent with the discussion above. The tumor cells cultured with Fe_3_O_4_-RGD can accumulate more Fe_3_O_4_ nanoclusters than that cultured with Fe_3_O_4_-DMSA. These results confirm that the RGD ligand can enable an active targeting effect toward tumor cells.

### T_1_/T_2_ dual-mode MR imaging of Fe_3_O_4_-DMSA and Fe_3_O_4_-RGD nanoclusters both in vitro and vivo

**Figure 3:**
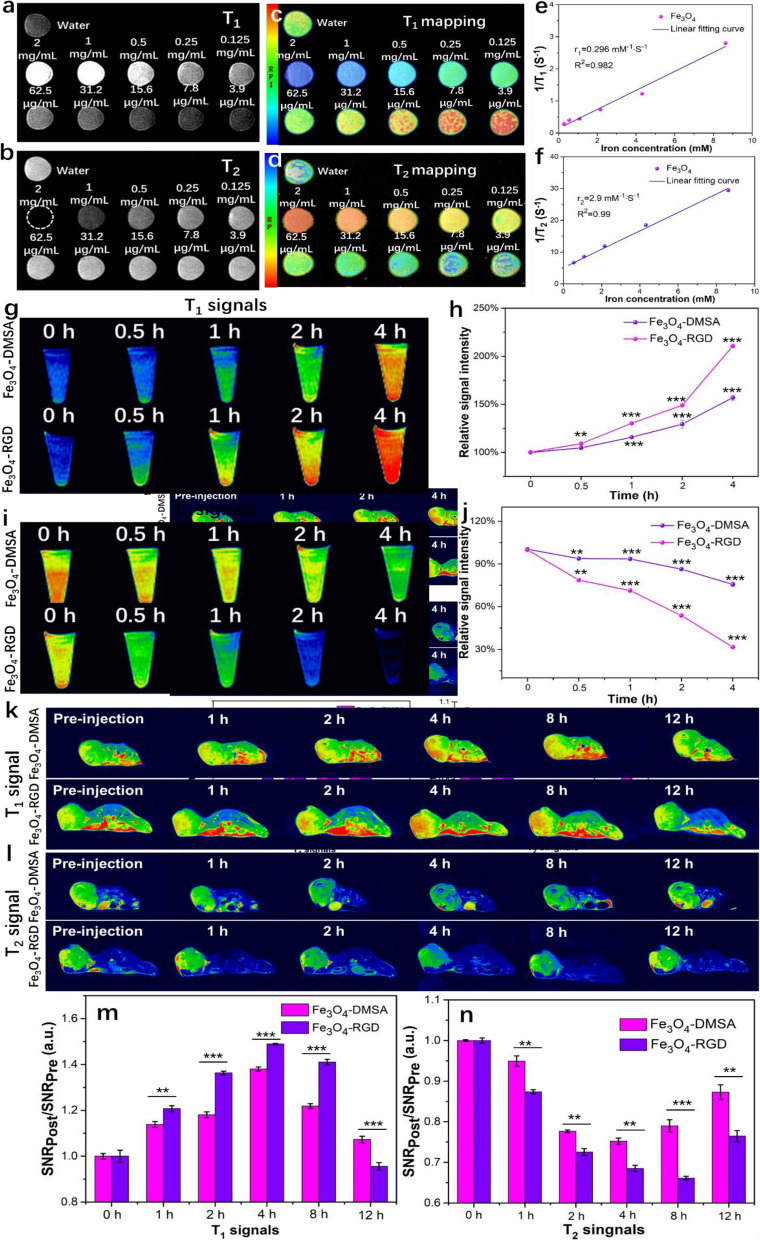
T_1_/T_2_ dual-mode MRI of Fe_3_O_4_-DMSA and Fe_3_O_4_-RGD nanoclusters both in vitro and vivo. (**a**) T_1_-weighted MR images, (**c**) T_1_ mapping and (**e**) T_1_ relaxation time of Fe_3_O_4_-DMSA nanoclusters at various iron concentrations. (**b**) T_2_-weighted MR images, (**d**) T_2_ mapping and (**f**) T_2_ relaxation time of Fe_3_O_4_-DMSA nanoclusters at various iron concentrations. (**g**) T_1_ MRI images of 4T1 cells cultured with Fe_3_O_4_-DMSA and Fe_3_O_4_-RGD nanoclusters and (**h**) the corresponding signal intensity statistics. (**i**) T_2_ MRI images of 4T1 cells cultured with Fe_3_O_4_-DMSA and Fe_3_O_4_-RGD nanoclusters and (**j**) the corresponding signal intensity statistics. (**k**) T_1_ MRI images of tumors in vivo at different times after the injection of Fe_3_O_4_-DMSA and Fe_3_O_4_-RGD nanoclusters. (**l**) T_2_ MRI images of tumors in vivo at different times after the injection of Fe_3_O_4_-DMSA and Fe_3_O_4_-RGD nanoclusters. (**m**) T_1_- and (n) T_2_-weighted MRI signal intensity statistics at different times.

The T_1_/T_2_ dual-mode MRI of Fe_3_O_4_-DMSA and Fe_3_O_4_-RGD nanoclusters both in vitro and in vivo were furtherly studied. As shown in Fig. 3a, for T_1_-weighted imaging, the phantom images show enhanced lightness with increasing Fe_3_O_4_ concentration. This result confirms that Fe_3_O_4_-DMSA nanoclusters possess the typical characteristics of T_1_ contrast agents. For T_2_-weighted imaging, the phantom images tend to be darker when the Fe_3_O_4_ concentration increases (Fig. 3b), which shows the typical characteristics of T_2_ contrast agents. The T_1_-weighted and T_2_-weighted MRI signals of the gradient concentrations of Fe_3_O_4_ nanoclusters indicate that the Fe_3_O_4_-DMSA nanoclusters is an excellent dual-mode T_1_ and T_2_ MRI contrast agent. The T_1_ and T_2_ relaxation times of the nanoclusters were simultaneously measured and are shown as T_1_ and T_2_ mappings according to the time horizon, respectively (Fig. 3c and d). The relaxivity values (r_1_ and r_2_) were obtained from the plots of inverse relaxation time (1/T_1_ and 1/T_2_) versus iron concentration (Fig. 3e and f). The calculated values of r_1_ and r_2_ are 0.296 mM^−1^ s^−1^ and 2.9 mM^−1^ s^−1^, respectively, and the r_2_/r_1_ value is 9.8. Generally, MRI contrast agents with a r_2_/r_1_ ratio of approximately 5–10 are determined to be T_1_/T_2_ dual-mode MRI contrast agents. Therefore, the synthesized Fe_3_O_4_-DMSA nanoclusters are excellent candidates of high-performance dual-mode contrast agents. The mechanism lies in that there still exist abundant Fe^3+^ ions on the surface of self-assembled nanocluster, which endow the excellent T_1_ mode MRI effect and the several nanoparticles self-assembled nanoclusters increase the equivalent volume of magnetic core, resulting in enhanced T_2_ mode MRI effect^24,35^.

Based on the RGD targeting effect, the MRI effect of the designed nanoprobes were investigated on 4T1 cells before practical application in vivo. 4T1 cells were cultured with Fe_3_O_4_-DMSA or Fe_3_O_4_-RGD nanoclusters at a concentration of 100 μg/mL for 0 h, 0.5 h, 1 h, 2 h, and 4 h. Then, the cells were digested, resuspended in PBS and investigated by the 3.0 T MR scanner. The T_1_-weighted images (Fig. 3g) show that the T_1_ MRI signals became stronger with culture time for 4T1 cells cultured with either Fe_3_O_4_-DMSA or Fe_3_O_4_-RGD. Thus, more Fe_3_O_4_ accumulates in 4T1 cells during the culture. The cells cultured with Fe_3_O_4_-RGD for various times showed a higher intensity of MRI response than those cultured with Fe_3_O_4_-DMSA at the same culture time because the targeting effect of RGD on 4T1 cells enhances the accumulation of Fe_3_O_4_ nanoclusters. The statistical intensity data were analyzed as shown in Fig. 3h. The T_1_ signal intensities were 104%, 115.7%, 129.3% and 157% in Fe_3_O_4_-DMSA and 109%, 130%, 149% and 210.6% in Fe_3_O_4_-RGD compared with the control group after culture with 4T1 cells for 0.5 h, 1 h, 2 h and 4 h, respectively. Since the T_2_ mode MRI is a negative mode, the contrast agent reduces the signal. Similar to the T_1_-weighted images, the T_2_ MRI signals (Fig. 3i) become weaker with increasing culture time for both 4T1 cells cultured with Fe_3_O_4_-DMSA and Fe_3_O_4_-RGD. The cells cultured with Fe_3_O_4_-RGD for various times show a weaker intensity of MRI response than those cultured with Fe_3_O_4_-DMSA at the same culture time. These results indicate a better T_2_ mode MRI response with Fe_3_O_4_-RGD nanoclusters, which confirms the positive effect of the active targeting effect of RGD on 4T1 cells. The intensities are analyzed as shown in Fig. 3j. The T_2_ signal intensities were 93.7%, 93.5%, 86.3% and 75.6% in Fe_3_O_4_-DMSA and 78.7%, 71.3%, 53.7% and 31.6% in Fe_3_O_4_-RGD compared with the control group after culture with 4T1 cells for 0.5 h, 1 h, 2 h and 4 h, respectively. The results show that the Fe_3_O_4_ nanoclusters can actually serve as dual-mode T_1_/T_2_ MRI contrast agents to largely strengthen the contrast at the cellular level. Moreover, the RGD ligand can remarkably increase the targeting effect of Fe_3_O_4_ nanoclusters to form a faster and more high-intensity MRI response.

These Fe_3_O_4_-based nanoprobes were further investigated with 4T1 tumor-bearing mice in vivo. As shown in Fig. 3k and l, the T_1_ and T_2_ MRI images were acquired at different times after the intravenous injection of the Fe_3_O_4_-DMSA and Fe_3_O_4_-RGD nanoprobes. The effect of the contrast agent on T_1_- and T_2_-weighted images increases and decreases the MRI response, which are shown in red and blue, respectively. For the T_1_ signals (Fig. 3k), the signal intensity at the tumor region in both Fe_3_O_4_-DMSA and Fe_3_O_4_-RGD groups gradually increases during the first 4 h due to the rapid accumulation of Fe_3_O_4_ nanoclusters but shows a slowed increasing tendency at the later time, which may be due to the metabolism in vivo and small accumulation amount of Fe_3_O_4_ nanoclusters. At 4 h, the tumor tissue shows the best contrast to the surrounding tissues, which enables the diagnosis of the tumor tissue to be more accurate. Moreover, compared to the effect of Fe_3_O_4_-RGD with Fe_3_O_4_-DMSA, due to the targeting function of RGD to 4T1 cells, the tumor tissue of the Fe_3_O_4_-RGD group shows a higher contrast to the surrounding tissues than that of the Fe_3_O_4_-DMSA group at the same MRI examination time. Moreover, the enhanced contrast lasts longer for the Fe_3_O_4_-RGD group. Thus, the RGD targeting effect reduces metabolism in vivo. For T_2_-weighted imaging (Fig. 3l), the contrast enhancement of the tumor tissue was almost identical to the T_1_ MRI result. The main difference may be that the T_2_ contrast enhancement in Fe_3_O_4_-RGD is more obvious over time, which may be due to the constant aggregation effect of Fe_3_O_4_ nanoclusters at the tumor site. These MRI results in vivo are mostly consistent with the in vitro results (Fig. 3g and i). To quantitatively analyze the contrast enhancement of T_1_ and T_2_ mode MRI, the signal intensity ratios SNRpost/SNRpre at different time points were analyzed and are shown in Fig. 3m and n, respectively. The T_1_ signal intensities in the tumor sites were 113.8%, 118.1%, 138.1%, 121.9% and 107.3% in the Fe_3_O_4_-DMSA group and 120.8%, 136.4%, 149%, 141% and 95.6% in the Fe_3_O_4_-RGD group compared with the control group after 1 h, 2 h, 4 h, 8 h and 12 h of intravenous injection, respectively. The T_2_ signal intensities in the tumor sites were 94.9%, 77.7%, 75.2%, 79% and 87.3% in the Fe_3_O_4_-DMSA group and 87.4%, 72.6%, 68.5%, 66.1% and 76.5% in the Fe_3_O_4_-RGD group compared with the control group after 1 h, 2 h, 4 h, 8 h and 12 h of intravenous injection, respectively. All of results confirm that Fe_3_O_4_-RGD can serve as an efficient activatable dual-mode MRI contrast agent for precise tumor diagnosis in vivo due to the ultra-small nanoparticle-based nanocluster structure and RGD targeting effect, which reveals its tremendous potential application for accurate tumor diagnosis in the clinic.

After the injection of the Fe_3_O_4_ nanocluster-based nanoprobe, the in vivo iron distribution and biosafety were further investigated. Frozen sections of the tumor tissue were stained with Prussian blue to reveal the Fe distribution. As shown in Fig. S7, without Fe_3_O_4_ injection, no Fe nanoparticles were present in the tumor tissue in the control. Some regions stained blue due to Fe accumulation after the intravenous injection of Fe_3_O_4_-DMSA. In contrast, large areas were stained blue with the intravenous injection of Fe_3_O_4_-RGD. This result further confirms the targeting effect of RGD and the induced improved accumulation property of Fe_3_O_4_-RGD. In addition, the Fe_3_O_4_-DMSA nanoclusters are dispersed in the tumor tissue with no mass aggregation, but most of the Fe_3_O_4_-RGD nanoclusters aggregate near the blood vessels, which indicates that the RGD ligand can efficiently target the α_v_β_3_ receptors of endothelial cells in the tumor site and tumor cells. Furthermore, ultrathin sections of tumor tissue were examined by TEM (Fig. S8). Fe_3_O_4_-based nanoclusters were detected at the tumor sites in both groups. To understand the metabolism of the Fe_3_O_4_ nanoclusters, the amount of Fe in the main organs and tumors in vivo was analyzed by ICP-MS measurements (Fig. S9). The Fe ion concentrations in the heart, spleen, lung and kidney hardly change with or without the intravenous injection of Fe_3_O_4_ nanoclusters. The Fe concentration significantly increases from ~ 100 to ~ 400 μg/g in the liver, which indicates that the liver is the main uptake organ and key metabolic organ for Fe_3_O_4_ nanoclusters. Notably, the Fe concentration in tumors increases with the application of Fe_3_O_4_ nanoclusters. Moreover, the Fe_3_O_4_-RGD group was almost 4 times the control and 3 times the Fe_3_O_4_-DMSA group. This result is attributed to the great targeting effect of RGD on 4T1 cells. Good hemocompatibility is essential for nanomaterials for biomedical applications in vivo. The hemocompatibility of the Fe_3_O_4_ nanocluster-based nanoprobe was evaluated through a hemolytic assay (Fig. S10). Compared with the effects of water (positive control) and physiological saline (negative control), no distinct hemolysis could be observed with either Fe_3_O_4_-DMSA or Fe_3_O_4_-RGD over the studied concentration range. The quantitatively analyzed hemolysis percentages of these nanoclusters were less than 2%, even when the concentration increased to 100 μg/mL These results suggest that the designed nanoprobe exhibits excellent hemocompatibility.

### Fe_3_O_4_ nanoclusters based nanomedicine for T_1_/T_2_ dual-mode MR imaging and synergistic CDT and chemotherapy

Before the Fe_3_O_4_ nanoclusters being used as the MRI contrast agent and chemotherapy drug carries of tumor in vivo, 4T1 cells were firstly cultured with Fe_3_O_4_-DMSA nanoclusters at different concentrations to evaluate the CDT effect. The living/dead staining results (Fig. S11) show that there were fewer living cells with increasing Fe_3_O_4_ concentration and culture time. The CCK-8 results (Fig. S12) show the same trend of growth inhibition and toxicity effects on the tumor cells. Moreover, the cytotoxicity of Fe_3_O_4_-RGD shows distinct differences (in the concentration of 50 μg/mL, p = 0.017; 100 μg/mL, p = 0.003 and 150 μg/mL, p < 0.001) compared with Fe_3_O_4_-DMSA. This result indicates that the RGD-modified nanoparticles potentiate the CDT effect by enhancing tumor targeting effect. The ROS level in the tumor cells was detected and stained after culture with Fe_3_O_4_ nanoclusters (Fig. S13). When cultured with 50 μg/mL Fe_3_O_4_, the ROS level was much higher than that of the control. In addition, it is furtherly enhanced with 150 μg/mL of Fe_3_O_4_ nanoclusters. Therefore, the apoptosis of tumor cells induced by a high degree of oxidative stress should account for the inhibitory and toxic effects of nanoclusters. Moreover, these results indicate the abundant exposed Fe ions of the ultra-small Fe_3_O_4_ nanoparticles play a key role for the CDT of tumor cells.

**Figure 4:**
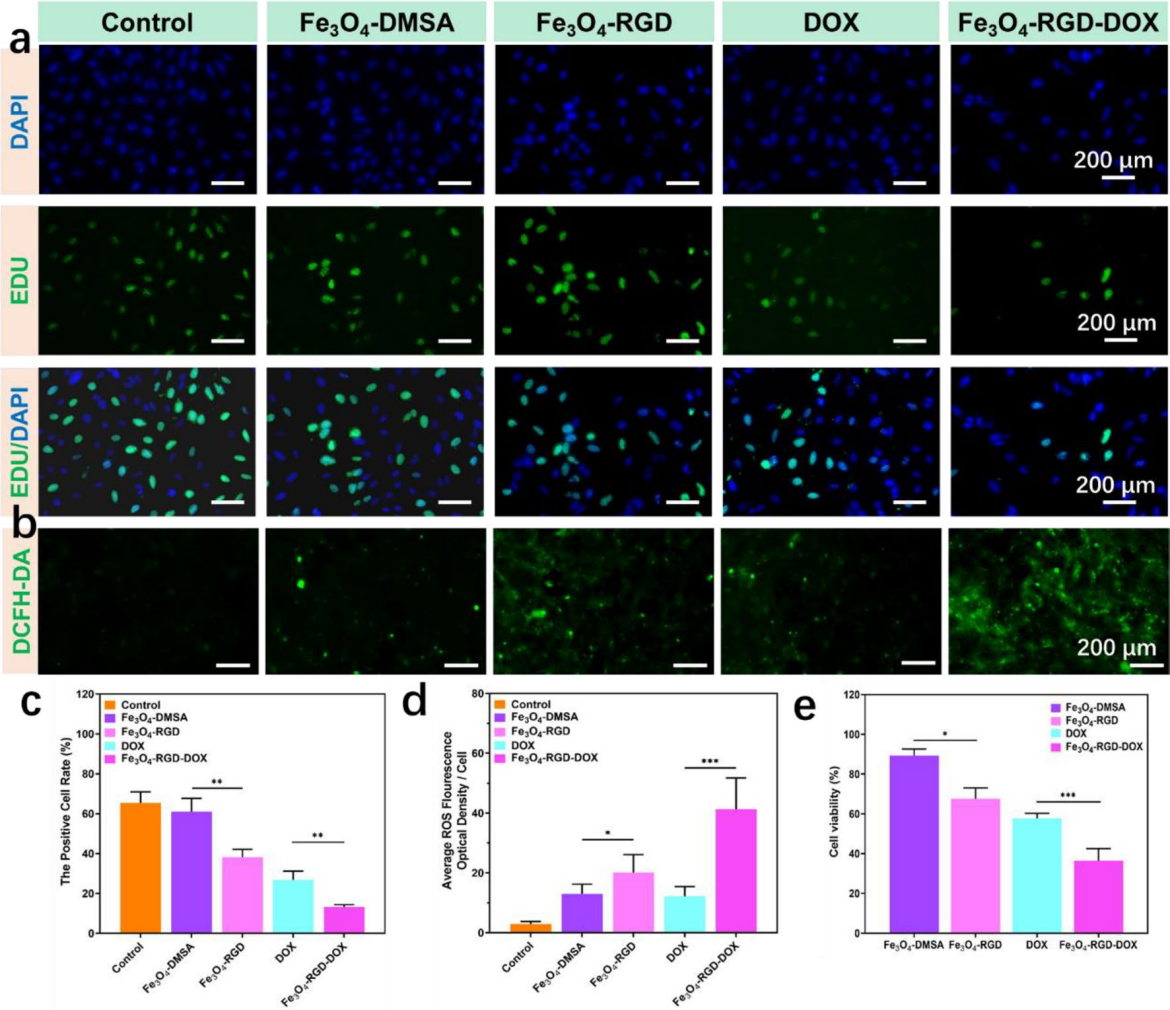
The biocompatibility of Fe_3_O_4_ nanoclusters based nanomedicine (Fe_3_O_4_-DMSA, Fe_3_O_4_-RGD, Fe_3_O_4_-RGD-DOX) in vitro. (**a**) EDU/DAPI staining results of 4T1 cells after coculturing with Fe_3_O_4_ nanoclusters based nanomedicine. (**b**) ROS staining results of 4T1 cells after coculturing with Fe_3_O_4_ nanoclusters based nanomedicine. (**c**) the statistical positive cell rate of 4T1 cells after being stained by EDU kit. (**d**) the average ROS fluorescence density statistics of 4T1 cells after being stained by DCFH-DA. (**e**) CCK-8 results of 4T1 cells after coculturing with Fe_3_O_4_ nanoclusters based nanomedicine.

After the conjugation of RGD ligand and DOX molecule on the surface of Fe_3_O_4_-DMSA nanoclusters, the integrated theranostic platform could endow the T_1_/T_2_ dual-mode MRI imaging guided synergistic CDT and chemotherapy of tumor cells. Fe_3_O_4_ nanoclusters based nanomedicine (Fe_3_O_4_-DMSA, Fe_3_O_4_-RGD, Fe_3_O_4_-RGD-DOX) were co-cultured with 4T1 cells in vitro, the EDU test was used to classify the effect of Fe_3_O_4_ nanoclusters based nanomedicine on 4T1 cells. The proliferative capacity of the treated cells (Fig. 4a) in proliferative phase were dyed green (EdU-positive) and nuclei were dyed blue. Further detailed analyses (Fig. 4c) indicate that the number of proliferating cells was least in the Fe_3_O_4_-RGD-DOX group when compared to all other groups, demonstrating that combination treatment strategy induced obvious suppressing effect on cell proliferation. Inhibitory effect in Fe_3_O_4_-RGD group on cell proliferation was more obvious than that of Fe_3_O_4_ alone (38.19 ± 3.9% vs. 61.05 ± 6.72%, *p < 0.05), indicating that RGD targeted ability was critical for combination therapy. In addition, cell proliferation in DOX group was higher than that of Fe_3_O_4_-RGD-DOX group (26.8 ± 4.4% vs. 13.2 ± 1.1%, **p < 0.01), indicating that Fe_3_O_4_-RGD-DOX suppresses cell proliferation by enhanced intracellular DOX aggregation and synergized CDT. Furtherly, the ROS staining of 4T1 cells treated with Fe_3_O_4_ nanoclusters based nanomedicines was done and shown in Fig. 4b. The statistical result of staining was shown in Fig. 4d. The results indicate that the group of DOX shows a certain degree of green fluorescence caused by oxidative stress in tumor cells. The green fluorescence rate in the group of Fe_3_O_4_-RGD was higher than group of Fe_3_O_4_-DMSA (12.99 ± 3.29% *vs* 20.01 ± 5.97%, **p < 0.01). This result confirms that RGD could enhance the intracellular Fe_3_O_4_ level content and the Fe_3_O_4_ nanoclusters further increase the intracellular ROS through Fenton-like reaction. Moreover, after incubation with Fe_3_O_4_-RGD-DOX, the ROS level is highest compared with other groups which indicates that Fe_3_O_4_-RGD-DOX possess synergistic effect in increasing intracellular ROS and enhancing the CDT distinctively. The CCK-8 test was also applied to assay the antitumor effect of the Fe_3_O_4_ nanoclusters based nanomedicine. As shown in Fig. 4e, the cell viability of 4T1 cells treated with Fe_3_O_4_-DMSA, Fe_3_O_4_-RGD, DOX and Fe_3_O_4_-RGD-DOX were 89.29 ± 3.32%, 67.6 ± 5.43%, 57.83 ± 2.44% and 36.47 ± 6.04% (n = 5), respectively. In addition, compared with that in the DOX group, a larger decrease of cell viability was observed in the Fe_3_O_4_-RGD-DOX group at the same concentration of DOX (P < 0.001). These results suggest that Fe_3_O_4_-RGD could synergize the DOX (chemotherapy) with RGD targeted CDT effect.

**Figure 5:**
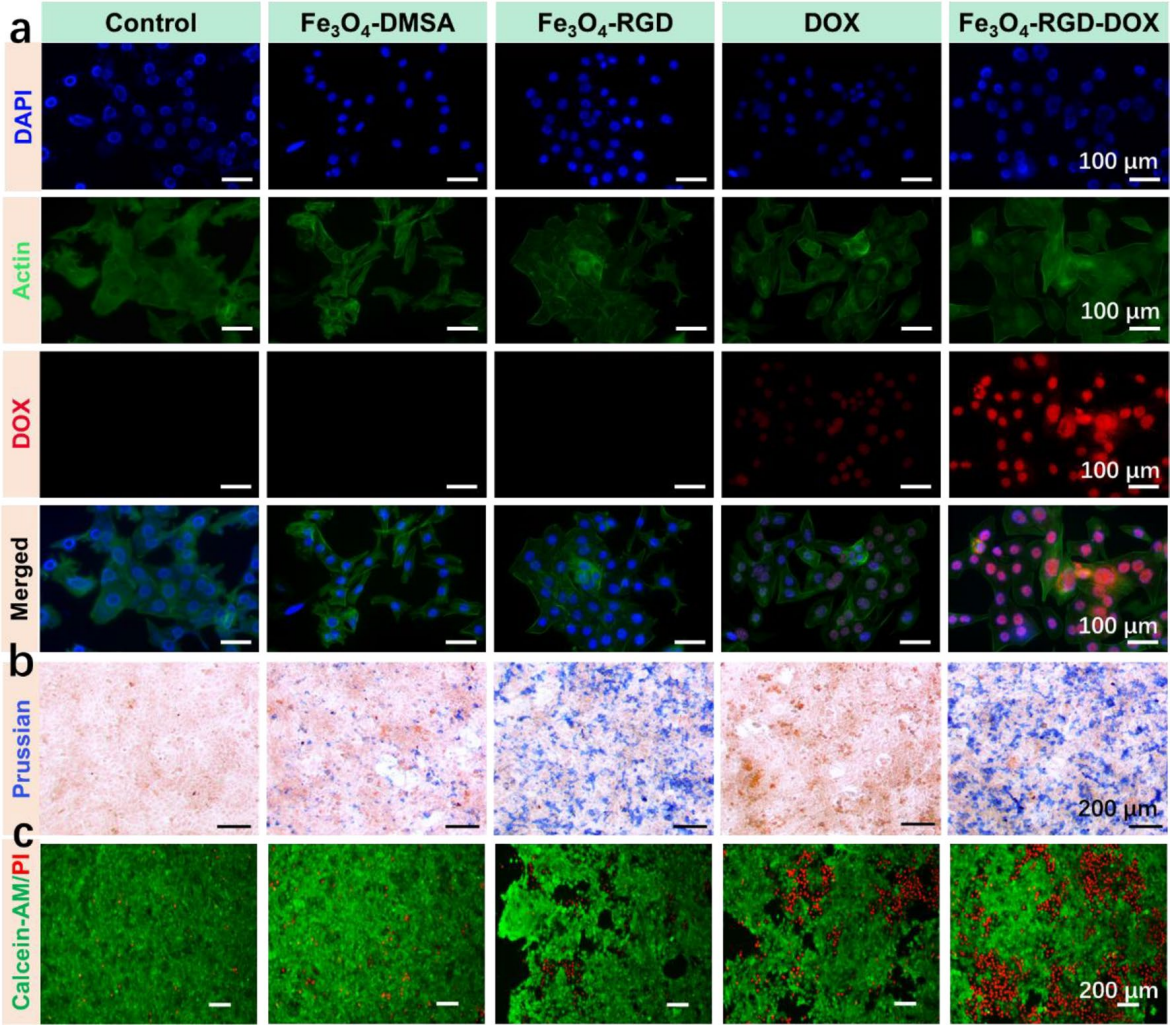
Synergistic CDT and chemotherapy effect of Fe_3_O_4_ nanoclusters based nanomedicine (Fe_3_O_4_-DMSA, Fe_3_O_4_-RGD, Fe_3_O_4_-RGD-DOX) in vitro. (**a**) DAPI/Actin/DOX staining results of 4T1 cells after culturing with Fe_3_O_4_ nanoclusters based nanomedicine. (**b**) Prussian blue staining results of 4T1 cells after coculturing with Fe_3_O_4_ nanoclusters based nanomedicine. (**c**) Living/dead staining results of 4T1 cells after culturing with Fe_3_O_4_ nanoclusters based nanomedicine.

To classify the synthesized tumor targeting effect and therapy effect, the DAPI/Actin/DOX staining (Fig. 5a) was conducted. The DAPI and the actin staining confirm the location of cell nucleus and cytoskeleton respectively. The DOX fluorescence mainly located at the cell nucleus and cytoplasm, which confirms the effective function of DOX molecule in the tumor cells. With the addition of RGD ligand, more DOX molecule could entry into cell nucleus and induce the apoptosis of tumor cells. To confirm the tumor targeted treatment of Fe_3_O_4_ nanoclusters based nanomedicine, the Prussian blue staining of 4T1 tumor cells cocultured with Fe_3_O_4_-DMSA, Fe_3_O_4_-RGD, DOX and Fe_3_O_4_-RGD-DOX. As shown in Fig. 5b, the Fe_3_O_4_-DMSA, Fe_3_O_4_-RGD and Fe_3_O_4_-RGD-DOX group show blue sediment due to the Fe distribution in tumor cells. Moreover, the Fe_3_O_4_-RGD and Fe_3_O_4_-RGD-DOX group show more obvious Fe sediment, which is due to the RGD targeting ability toward 4T1 cells. The living/dead staining were conducted and the results (Fig. 5c) indicated that the both the Fe_3_O_4_ nanoclusters and DOX could induce the death of tumor cells. Furtherly, the RGD ligand could enhance the killing effect of nanomedicine, which endow Fe_3_O_4_-RGD-DOX effective tumor targeted killing rate by synergistic CDT and chemotherapy.

**Figure 6:**
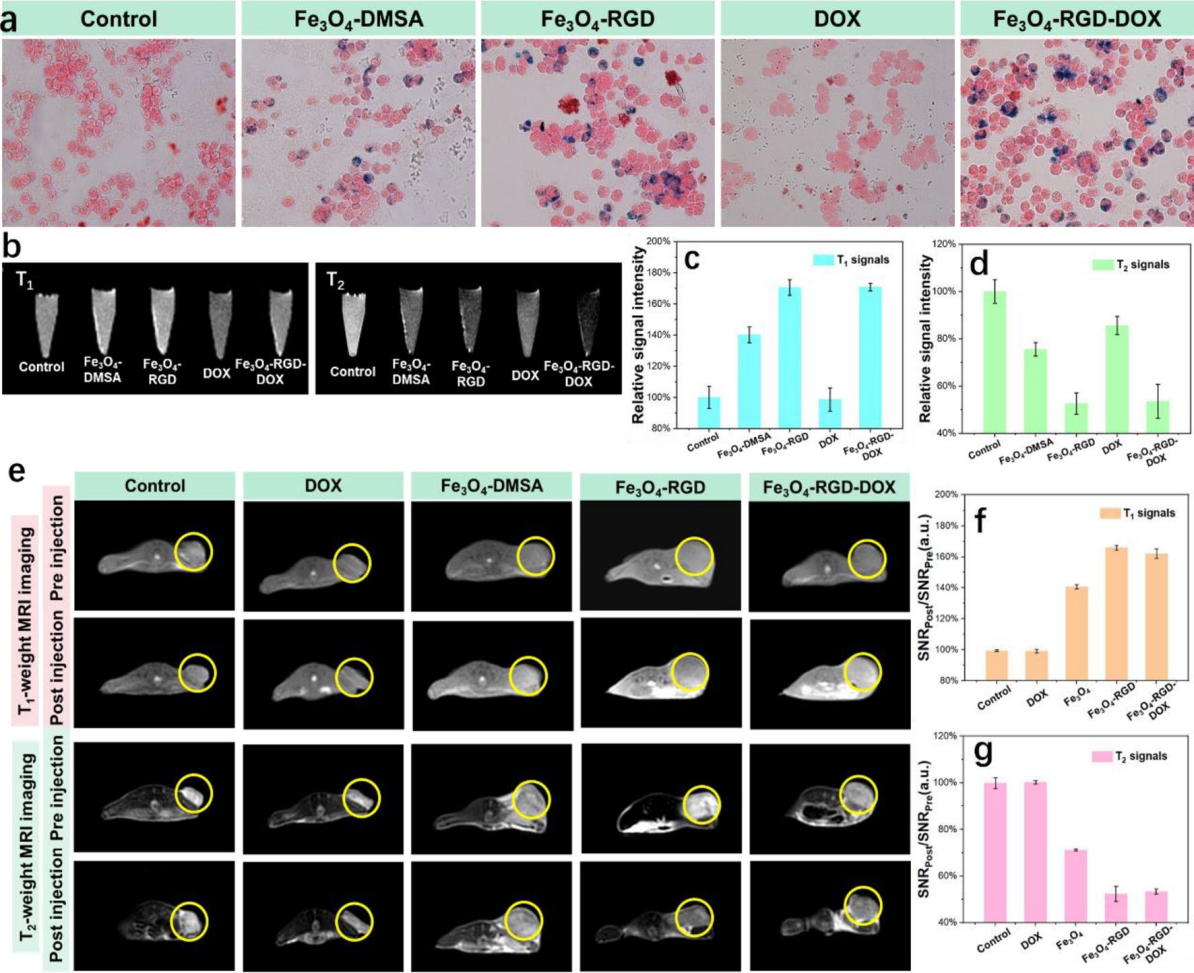
Tumor targeted T_1_ and T_2_ MRI images of tumor cells and tumors as well as the signals statistic. (**a**) Prussian blue staining, (**b**) The T_1_ and T_2_ MRI image and (**c**), (**d**) the corresponding MRI signals statistic of digested 4T1 cells after incubation with Fe_3_O_4_ nanoclusters based nanomedicine. (**e**) The T_1_ and T_2_ MRI image and (**f**,**g**) the corresponding MRI signals statistic of tumor tissue before and after intravenous injection of Fe_3_O_4_ nanoclusters based nanomedicines.

The MR image of tumor cell and tumor tissue endowed by Fe_3_O_4_ nanoclusters based nanomedicine was primarily conducted. As shown in Fig. 6a, the 4T1 cells were treated with Fe_3_O_4_ nanoclusters based nanomedicine and then digested, the treated tumor cells were further stained with Prussian blue. The staining result confirms the tumor targeting ability of RGD ligand and the DOX conjugation possesses no effect on the targeting effect. Furtherly, the digested tumor cells were imaged with both T_1_ and T_2_ weighted MR (Fig. 6b) and the signal statistics were shown in Fig. 6c and d. The nanomedicines induce distinctive enhancement of MRI signal intensity in both T_1_ and T_2_ MRI modal. Before injection of the designed nanomedicine, hemolysis test of Fe_3_O_4_ nanoclusters based nanomedicine was conducted. The results show that the nanomedicine possess excellent blood compatibility (Fig. S14). To confirm the MR guided combination therapy effect of CDT and chemotherapy in vivo, T_1_ and T_2_ weighted MR images were detected before and after intravenous injection of Fe_3_O_4_ nanoclusters based nanomedicine. As shown in Fig. 6e, compared with the control group, there was no significant difference in T_1_ and T_2_ imaging effects in the group of free DOX molecule before and after injection; The T_1_ weighted imaging signal intensity in tumor tissue increased in other groups, and the tumor tissue became brighter; For T_2_ imaging, the tumor tissue darkens and the signal intensity decreases. More accurately, as shown in Fig. 6f, the T_1_ imaging signals in Fe_3_O_4_-RGD and Fe_3_O_4_-RGD-DOX groups show significant increase (166% and 162%) compared with the Fe_3_O_4_-DMSA group (140%). As shown Fig. 6g, the T_2_ imaging signals in Fe_3_O_4_-RGD and Fe_3_O_4_-RGD-DOX groups show significant decrease (52% and 53%) compared with the Fe_3_O_4_-DMSA group (71%). These results indicate that RGD modified nanoclusters can enhance the effective aggregation of nanomaterials in tumor tissue through active targeted action, thereby further increasing the concentration of drugs in tumors. Meanwhile, the above results indicate that the Fe_3_O_4_-RGD-DOX nanocarrier platform can achieve visual evaluation of the treatment process through real-time in vitro MR monitoring.

**Figure 7:**
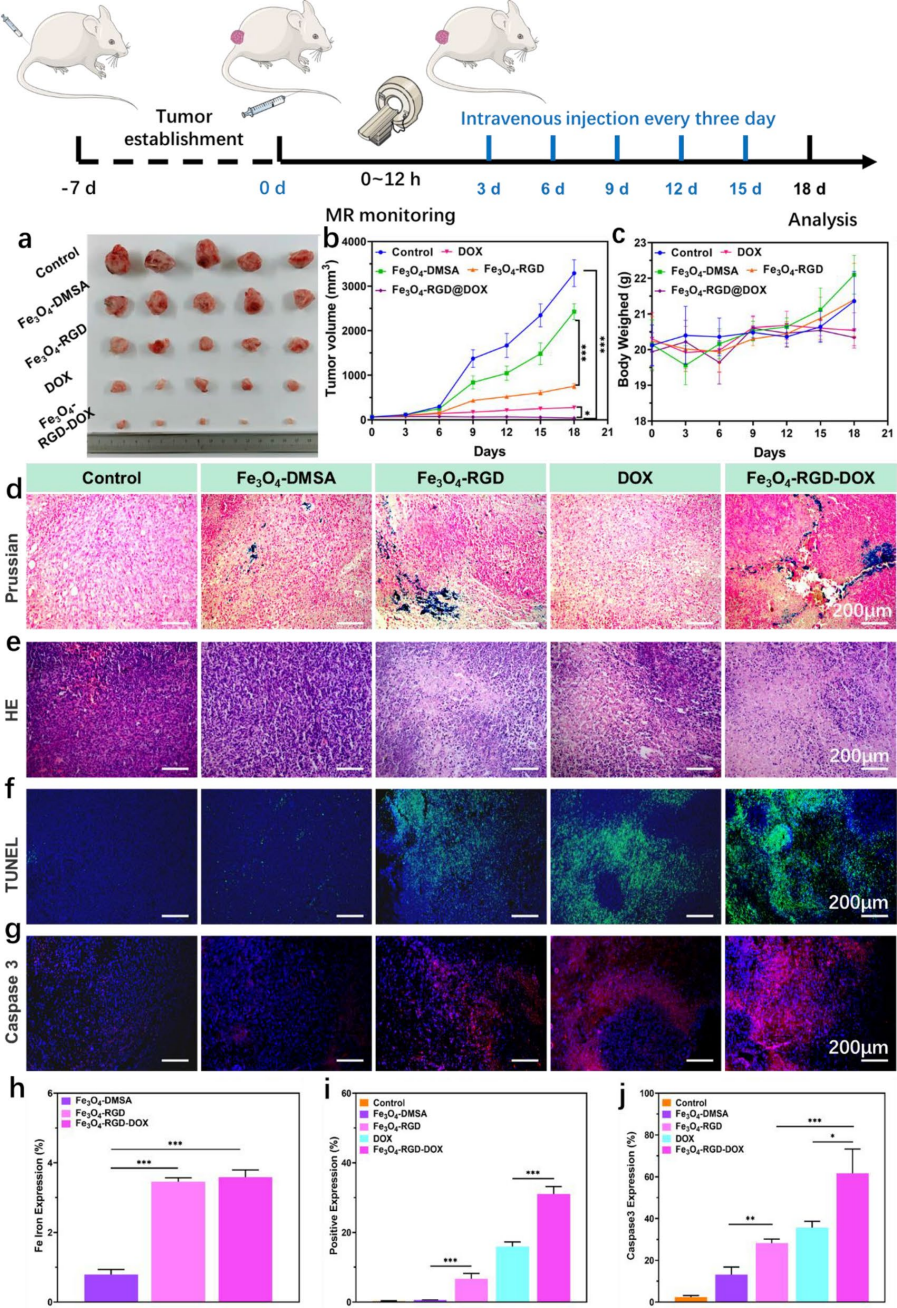
In *vivo* antitumor and biosafety evaluation of tumor targeted synergistic CDT and chemotherapy. (**a**) Real tumor image after the collaborative treatment. (**b**) Tumor volume of tumor bearing mice during the collaborative treatment. (**c**) Body weight of tumor bearing mice during the collaborative treatment. (**d**) Prussian blue staining, (**e**) HE staining, (**f**) TUNEL staining, (**g**) Caspase 3 staining of tumor tissue sections after the collaborative treatment. (**h**) The statistics of Fe ion expression obtained from Prussian blue staining (**i**) the statistics of positive expression of TUNEL. (**j**) The statistics of positive expression of Caspase 3.

The antitumor ability of Fe_3_O_4_-RGD-DOX based tumor targeted synergistic CDT and chemotherapy was evaluated in 4T1 tumor bearing BARB/C mice. The diagrammatic sketch of Fig. 7 detailly describes the treatment strategy. The tumor treatment results including tumor size and volume variety were shown in Fig. 7a and b. The results show that the Fe_3_O_4_-DMSA and Fe_3_O_4_-RGD groups show better tumor inhibition effect compared with the control group owing to the Fe_3_O_4_ mediated CDT in the tumor tissue. Moreover, the Fe_3_O_4_-RGD group exhibits more obvious inhibited effect compared with the Fe_3_O_4_-DMSA group (p < 0.001) owing to the tumor targeting effect of RGD ligand, which suggests that RGD mediated active targeting could enhance the CDT of Fe_3_O_4_. Compared with free DOX, the Fe_3_O_4_-RGD-DOX treated group exhibit significantly inhibition effect of tumor growth via the combinational effect from the chemotherapy and CDT. In addition, as shown in the Fig. 7c, the adopted treatment for mice would not influence the body weight, indicting the suitable dose and strategy of tumor therapy. In addition, the Prussian blue staining (Fig. 7d) and statistical result (Fig. 7h) show that the group of Fe_3_O_4_-RGD and Fe_3_O_4_-RGD-DOX have more Fe accumulation in tumor tissue. Hematoxylin/eosin (H&E) (Fig. 7e) and terminal deoxynucleotidyl transferase (TdT) dUTP nick-end labeling (TUNEL) staining with statistical result (Fig. 7f and i) show significant tumor cell death and the formation of many cavities, indicating that the Fe_3_O_4_-RGD-DOX can effectively induce tumor tissue damage. Meanwhile, the immunhistochemical studies (Fig. 7g and j) manifest that Fe_3_O_4_-RGD-DOX led to the most caspase3 expression, indicating that Fe_3_O_4_-RGD-DOX could efficiently induce tumor cell apoptosis, which well supported the in vitro anticancer result. All in all, these results indicated that the RGD meditated tumor targeted synergistic treatment effect of chemotherapy and CDT induced by ultrasmall Fe_3_O_4_ nanoclusters and DOX hold great tumor-growth inhibition effect.

### Biosafety evaluation after synergetic theranostics

**Figure 8:**
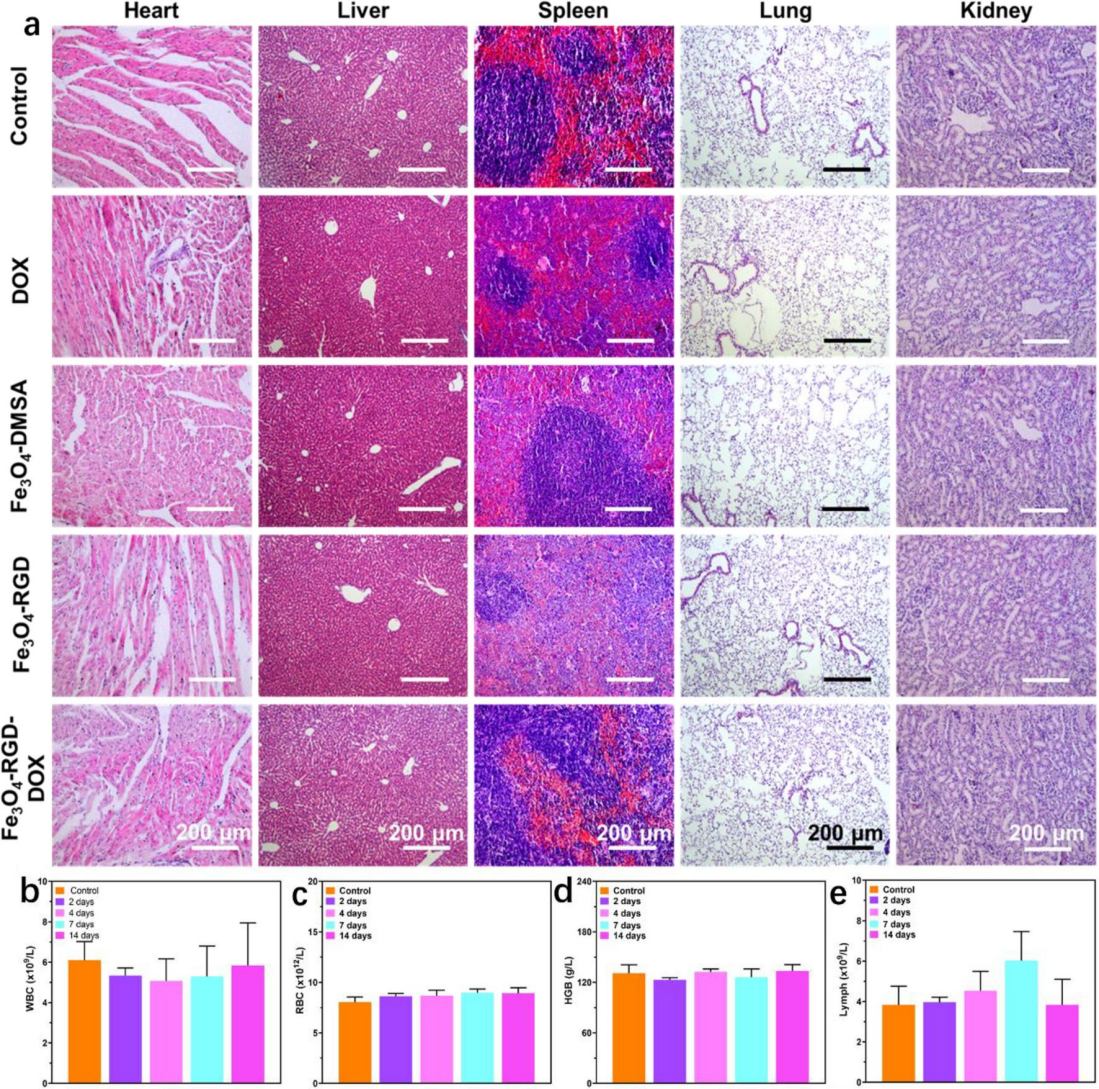
Biosafety evaluation of collaborative treatment of Fe_3_O_4_ nanoclusters based nanomedicine. (**a**) Images of hematoxylin and eosin (H&E)-stained histological tissue sections of major organs. (**b**–**e**) Main blood indexes of mice during the collaborative treatment.

After the in vivo antitumor study, the H&E staining of main organs including heart, liver, spleen, lung and kidney were used to evaluated biocompatibility and histological variations of Fe_3_O_4_ nanoclusters based therapy. As shown in Fig. 8a, the sections from all organ tissues in Fe_3_O_4_-DMSA, Fe_3_O_4_-RGD, DOX and Fe_3_O_4_-RGD-DOX groups demonstrated no histological difference compared with the control group. In addition, no histopathological damage was observed, including necrosis, swelling, or inflammatory response. Good hemocompatibility is also essential for the application of nanomaterials in biomedical applications in vivo. The hemocompatibility of the Fe_3_O_4_ nanocluster-based nanomedicine was evaluated through blood test. Moreover, the main blood indexes of mice treated with Fe_3_O_4_-RGD-DOX as well as other treatment at different days were further analyzed, showing no obvious change in all groups (Fig. 8b–e). These preliminary results show that the designed nanomedicines possess excellent biocompatibility in vivo.

## Conclusion

In summary, an effective method of mass produce ultra-small Fe_3_O_4_ nanoparticles was proposed. The further surface modification with DMSA endows the hydrophilic property and high colloidal stability in suspension as well as the self-assembled property. The obtained Fe_3_O_4_ nanoclusters show an excellent T_1_/T_2_ dual-mode MRI capacity and excellent potential for use as contrast agents due to the appropriate size effect. In addition, the ultrasmall Fe_3_O_4_ nanoclusters possess notable CDT effect caused by the increased ROS level in tumor cells. The collaborative work of chemotherapy drugs DOX and Fe_3_O_4_ nanoclusters base CDT treatment endow highly effective antitumor activity for tumor treatment. With the assistance of RGD targeting ligand, the integrated Fe_3_O_4_-RGD-DOX nanoplatform possesses tumor targeted T_1_/T_2_ dual-mode MRI guided synergistic CDT and chemotherapy, which provides a novel practical alternative for integrated diagnosis and treatment of tumors and exhibits promising potential for clinical translation.

### Supplementary Information

Supplementary Figures.

## Data Availability

The datasets used and/or analysed during the current study available from the corresponding author on reasonable request.
