# Supplementary material for: Ultrasmall Fe3O4 nanoparticles self-assembly induced dual-mode T1/T2-weighted magnetic resonance imaging and enhanced tumor synergetic theranostics
Source: Sci Rep. 2024 May 9;14:10646. doi: 10.1038/s41598-024-59525-2 (PMC11082189; doi:10.1038/s41598-024-59525-2)
Supplement: Supplementary file 1 — Supplementary Figures. [file 41598_2024_59525_MOESM1_ESM.docx]

**Supporting information of**

**Ultrasmall Fe_3_O_4_ nanoparticles self-assembly induced dual-mode T_1_/T_2_-weighted magnetic resonance imaging** **and** **enhanced tumor synergetic theranostics**


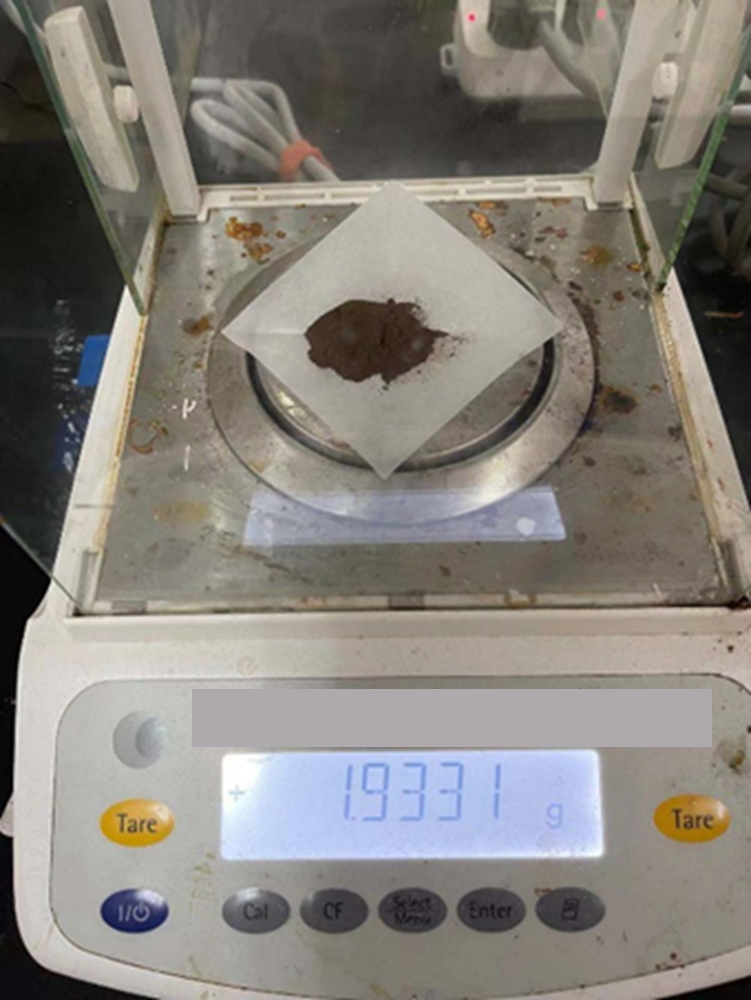


**Figure S1.** The mass-production of ultrasmall Fe_3_O_4_ nanoparticles.

The synthesized ultrasmall Fe_3_O_4_ nanoparticles through solvothermal method receive a mass-production of 1.9331 g in one reaction. The synthesis procedure could be magnified with more times to obtain more production.


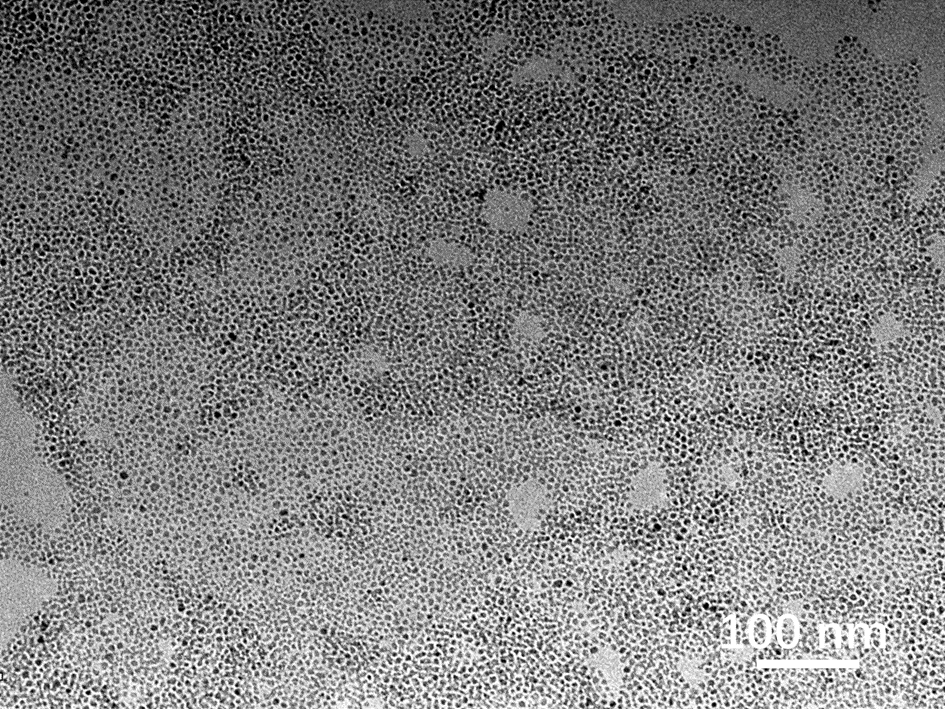


**Figure S2.** TEM image of ultrasmall Fe_3_O_4_ nanoparticles under low magnification.

The TEM image of ultrasmall Fe_3_O_4_ nanoparticles shows that the obtained nanoparticles are of uniform size on the whole and the main size distribution are around 5 nm.


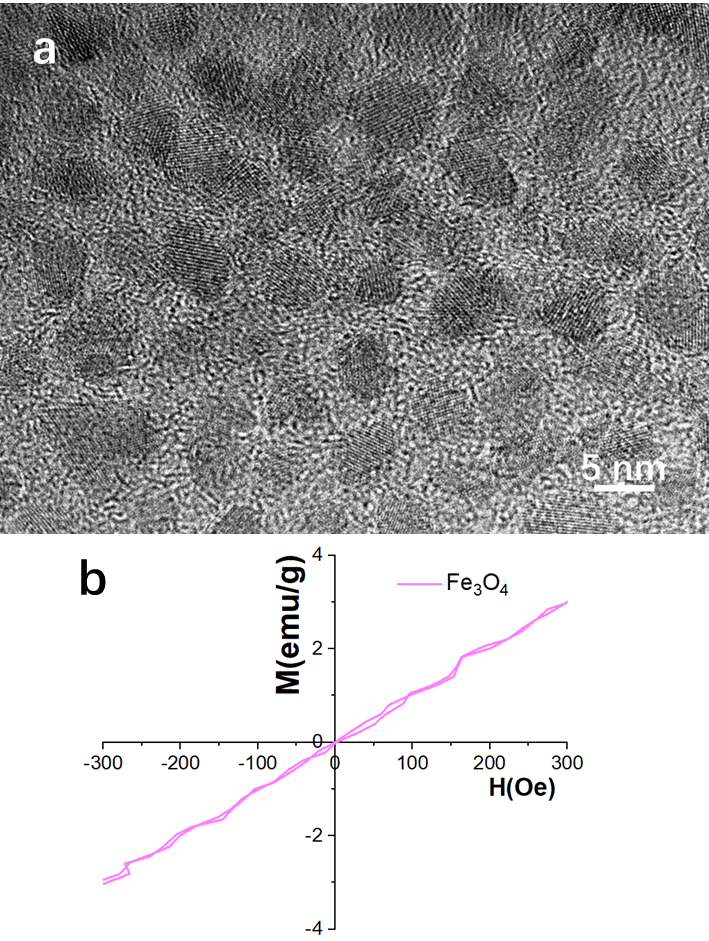


**Figure S3.** **(a)** HRTEM image of ultrasmall Fe_3_O_4_ nanoparticles in large vision. **(b)** enlarged M-H curve of ultrasmall Fe_3_O_4_ nanoparticles.

HRTEM image of ultrasmall Fe_3_O_4_ nanoparticles (Figure S3a) in large vision indicates that the nanoparticles are of good crystallinity, which endow the large saturation magnetization. Enlarged M-H curve (Figure S3b) proves the superparamagnetic property of ultrasmall Fe_3_O_4_ nanoparticles.


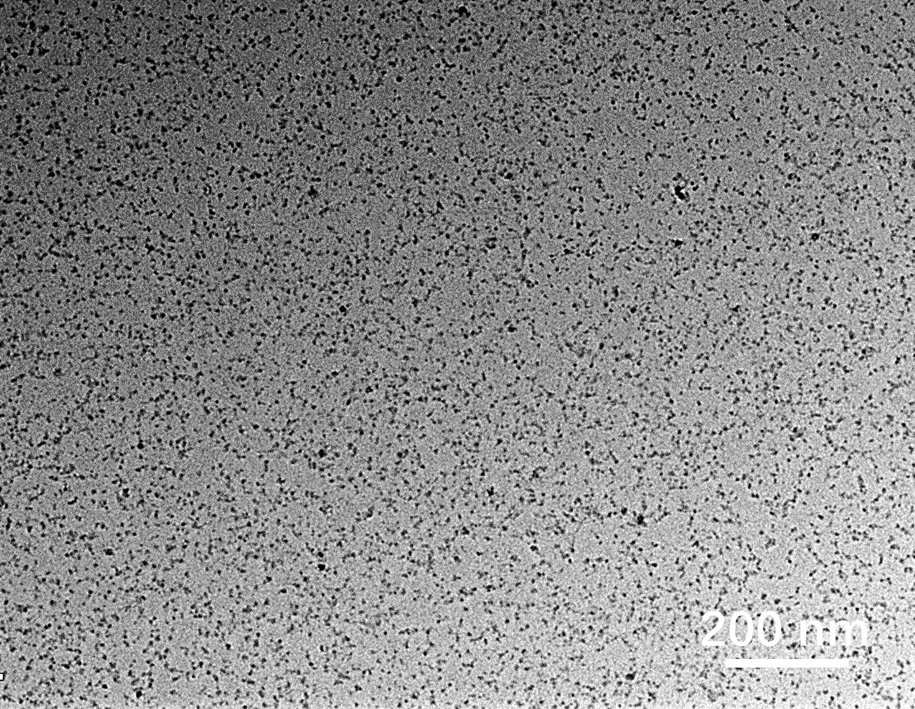


**Figure S4.** TEM image of Fe_3_O_4_-DMSA nanoclusters under low magnification.

The TEM image of Fe_3_O_4_-DMSA nanoclusters under low magnification indicates that the DMSA molecule modified Fe_3_O_4_ nanoparticles self-assembled into nanoclusters during the modification process, and the morphology keeps uniform on the whole, which endow the excellent water dispersity. The clustering mechanism lays that the carboxyl in DMSA molecule could coordinate with Fe ions on the surface of ultrasmall Fe_3_O_4_ nanoparticles. Under the ultrasonication treatment, ultrasmall Fe_3_O_4_ nanoparticles dispersed in solvent are modified with DMSA molecule and furtherly bonded with each other, inducing the formation of several nanoparticles contained nanoclusters.


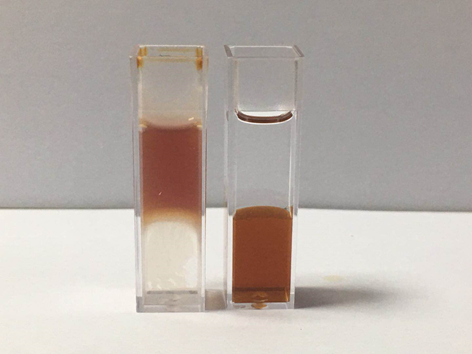


**Figure S5.** Phase transformation of Fe_3_O_4_ nanoparticles from n-hexane (upside) to water (downside) after the modification of DMSA molecule.

The DMSA could successfully modify the ultrasmall Fe_3_O_4_ nanoparticles and thus endow excellent water dispersity.


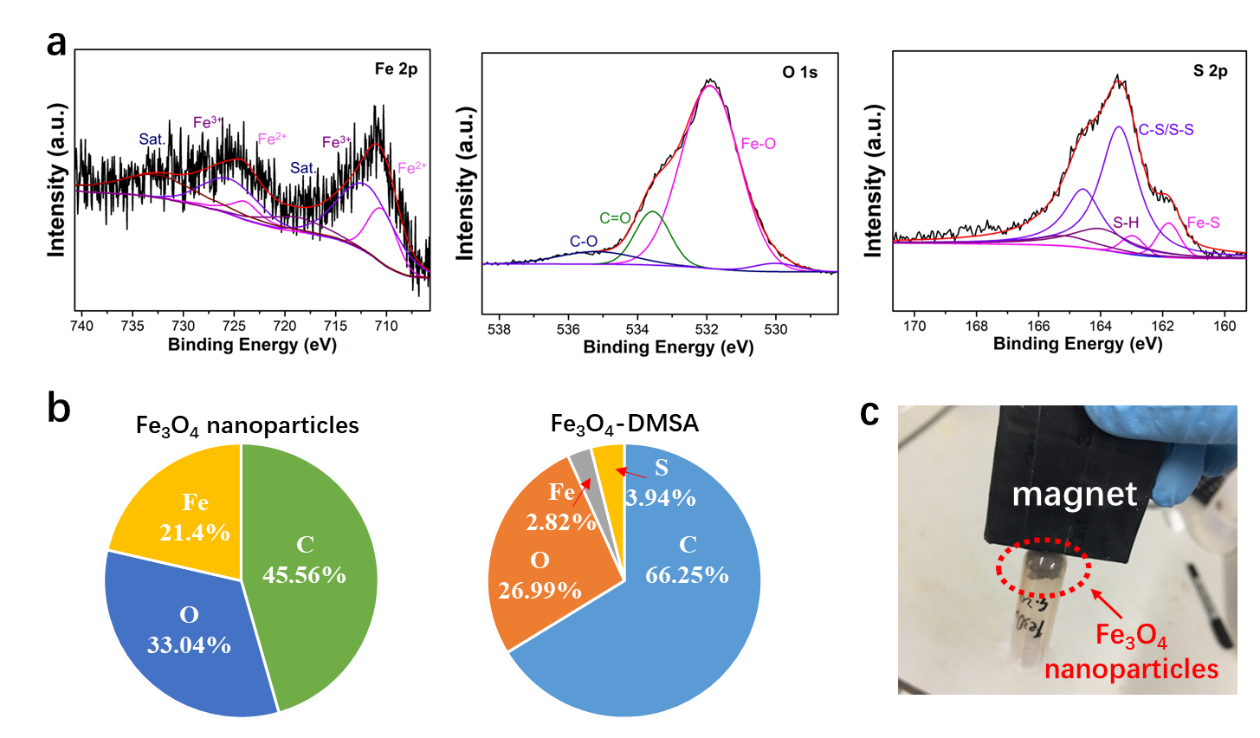


**Figure S6.** **(a)** atomic valence state analysis of Fe_3_O_4_-DMSA nanoclusters by XPS Fe 2p scan, O 1s scan and S 2p scan. **(b)** elemental content of ultrasmall Fe_3_O_4_ nanoparticles and Fe_3_O_4_-DMSA nanoclusters. **(c)** the ultrasmall Fe_3_O_4_ nanoparticles could be attracted by magnet.

The XPS pattern (Figure S6a) of Fe_3_O_4_-DMSA nanoclusters was analyzed. For the Fe 2p scan, the exit peaks of Fe^2+^ and Fe^3+^, indicating that the crystal structure are still Fe_3_O_4_ after DMSA modification. For the O 1s scan, there exist Fe-O, C=O, C-O, indicating the Fe_3_O_4_ and Carboxyl group of DMSA. For the S 2p scan, there exist S-H, C-S/S-S, indicating the sulfydryl group, and the Fe-S, demonstrating the partial bonding of iron and sulfur atom on the surface of Fe_3_O_4_ nanoparticles. The Figure S6b shows the exact element content of ultrasmall Fe_3_O_4_ nanoparticles and Fe_3_O_4_-DMSA nanoclusters. The Figure S6c shows the magnetic property of ultrasmall Fe_3_O_4_ nanoparticles.


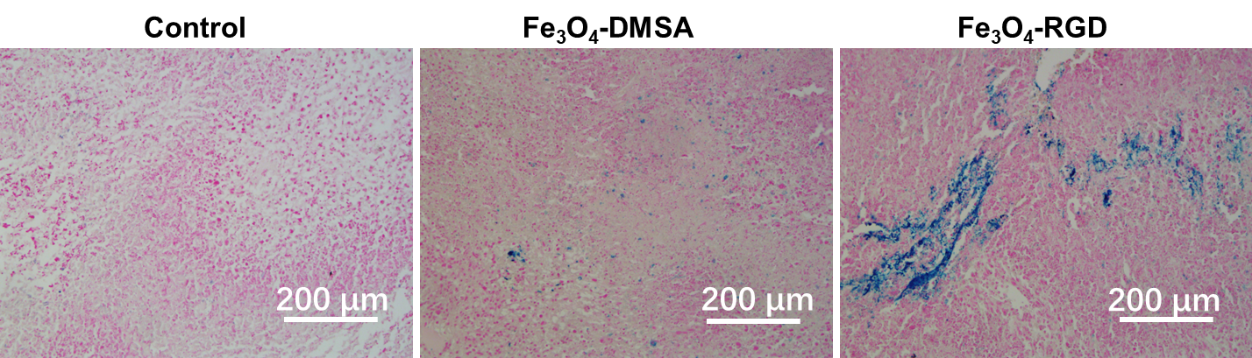


**Figure S7.** Prussian blue staining of tumor tissue sections after intravenous injection of Fe_3_O_4_ based nanoclusters.

The Prussian blue staining indicates the existence of Fe element. Therefore, as shown in the results above, the Fe_3_O_4_ based nanoclusters could serve as the MRI contrast agent in the tumor. Moreover, the RGD ligand could enhance the tumor targeted MRI effect of Fe_3_O_4_ nanoclusters.


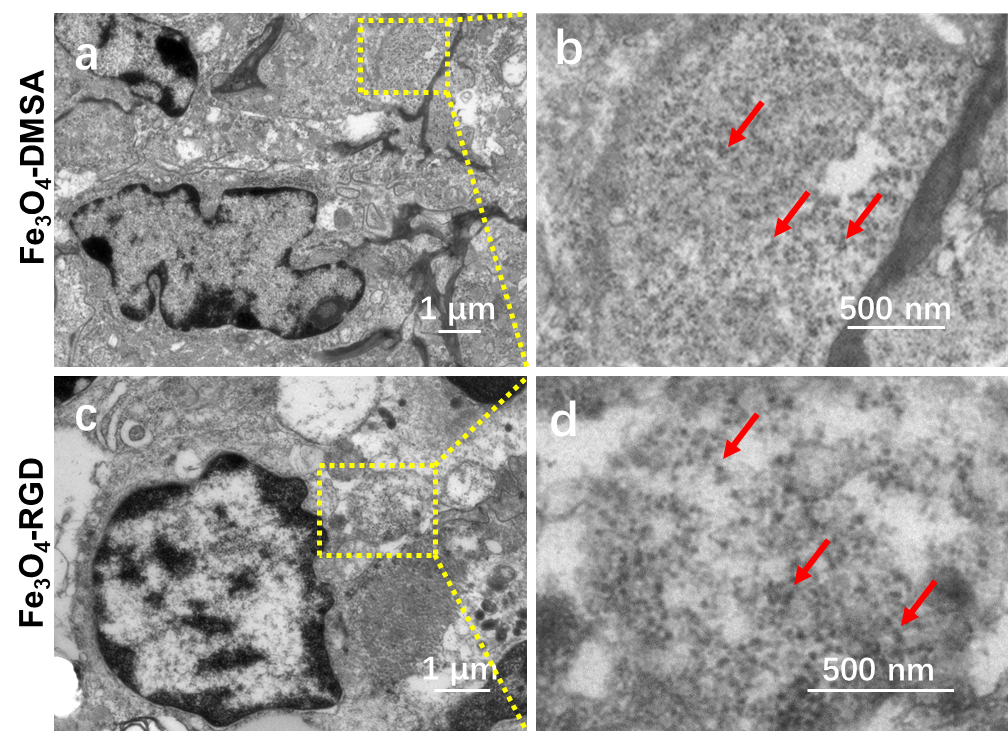


**Figure S8.** TEM images of tumor tissue sections and enlarged images of lysosomes.

As shown in the TEM images of tumor tissue sections and enlarged images of lysosomes, the Fe_3_O_4_ based nanoclusters could locate in the tumor cells by endocytosis.


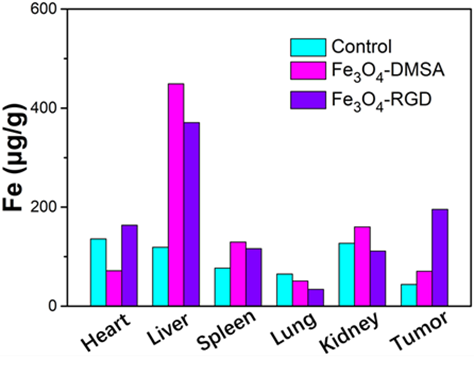


**Figure S9.** Iron distribution in the main organs after the injection of Fe_3_O_4_-DMSA and Fe_3_O_4_-RGD nanoclusters.


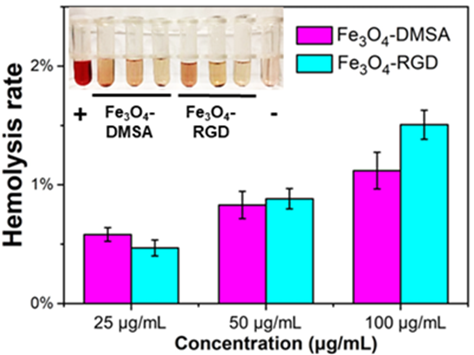


**Figure S10.** Hemolysis test of Fe_3_O_4_-DMSA and Fe_3_O_4_-RGD nanoclusters.


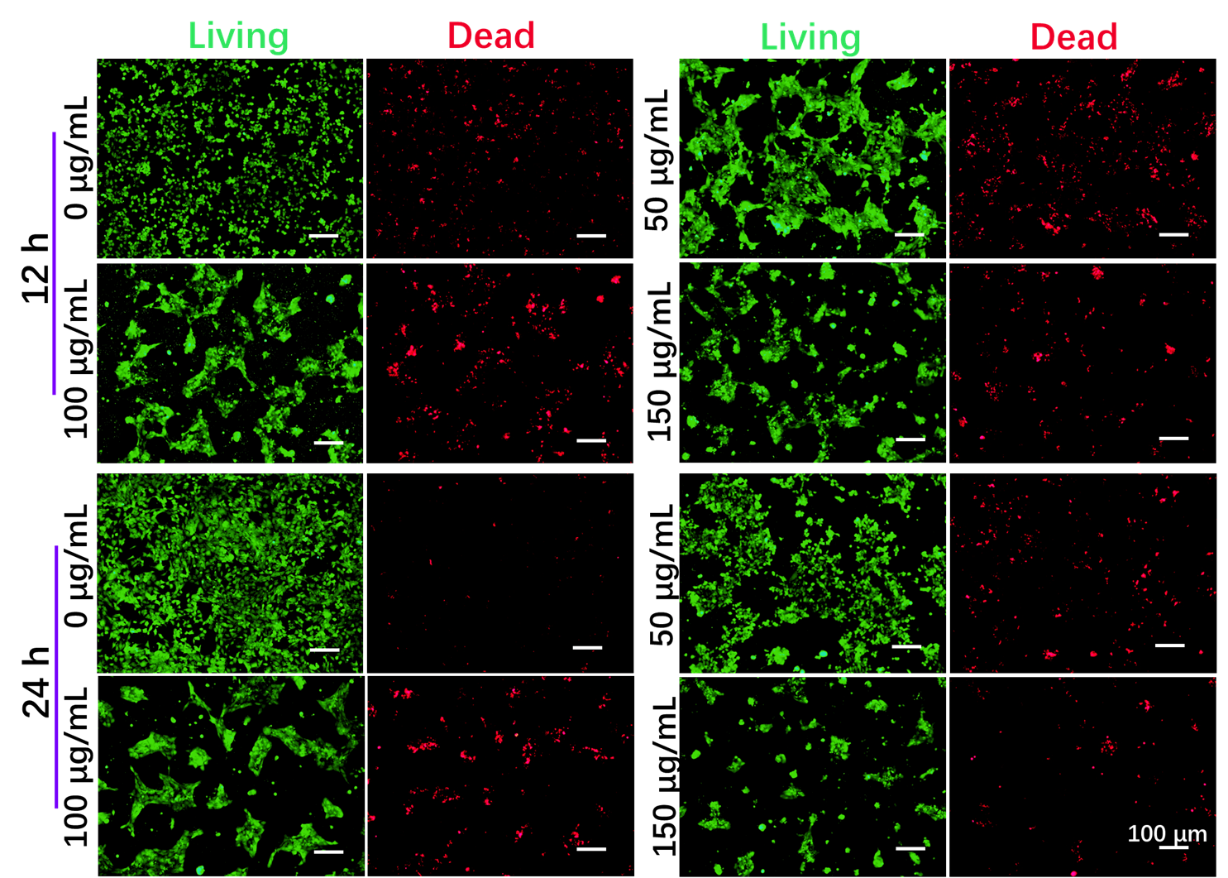


**Figure S11.** Living/dead staining of 4T1 cells cocultured with Fe_3_O_4_ nanoclusters with detailed information.

The living 4T1 cells were stained with green color and the dead cells were stained with red color. From the result, the 4T1 cells rapidly proliferate without the existence of Fe_3_O_4_ nanoclusters. When the nanoclusters were incubated with 4T1 cells, the proliferation rate was largely limited and the death rate increased too. Furthermore, with the increase of Fe_3_O_4_ nanoclusters in concentration and incubation time, the growth inhibition and death rate go stronger. This result demonstrates that the obtained Fe_3_O_4_ nanoclusters have obvious toxicity to the cancer cells.


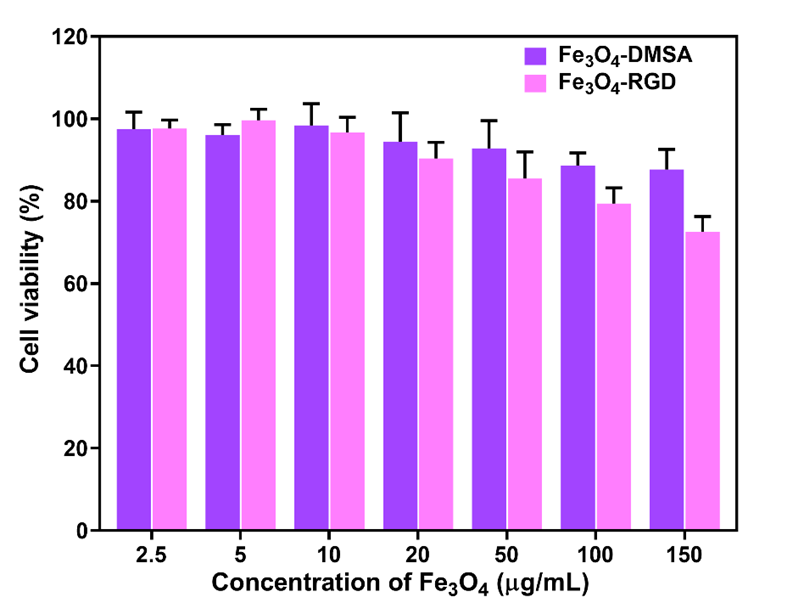


**Figure S12.** CCK-8 result of the coculture of Fe_3_O_4_ based nanoclusters and 4T1 cells.

With the increased concentration of Fe_3_O_4_-DMSA and Fe_3_O_4_-RGD nanoclusters, the cell activity decreased obviously. In addition, the conjugation of RGD ligand obviously enhance the decreasing rate of Fe_3_O_4_ nanoclusters.


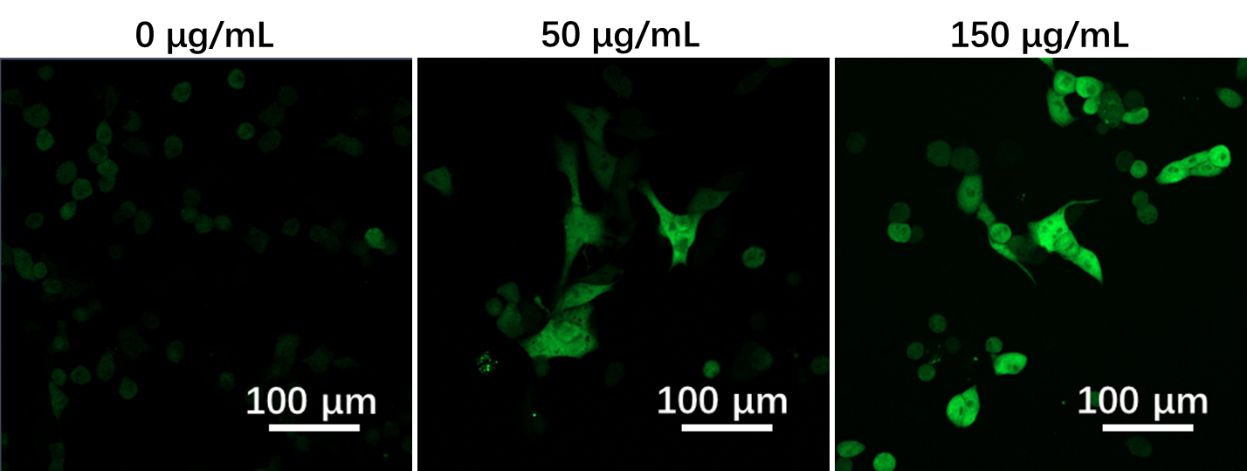


**Figure S13.** ROS staining of 4T1 cells after culture with different concentrations of Fe_3_O_4_ nanoclusters for 2 h.

The Fe_3_O_4_ nanoclusters could increase the ROS level in the 4T1 cells, which could induce the apoptosis of tumor cell.


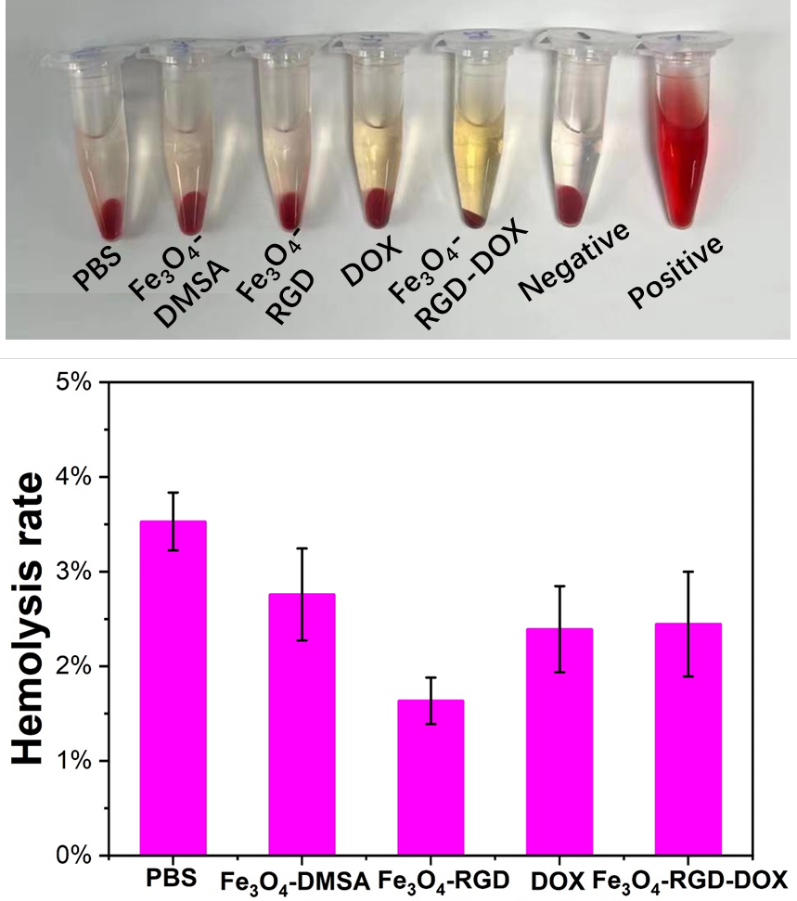


**Figure S14.** Hemolysis test of Fe_3_O_4_ nanoclusters and nanomedicine (200μg/mL).
